# Comprehensive evaluation of ibuprofenate amino acid isopropyl esters: insights into antioxidant activity, cytocompatibility, and cyclooxygenase inhibitory potential

**DOI:** 10.1007/s43440-024-00666-6

**Published:** 2024-10-19

**Authors:** Magdalena Perużyńska, Anna Nowak, Anna Muzykiewicz-Szymańska, Łukasz Kucharski, Joanna Klebeko, Karolina Bilska, Ewelina Kopciuch, Radosław Birger, Marek Droździk, Paula Ossowicz-Rupniewska

**Affiliations:** 1 Department of Experimental and Clinical Pharmacology, Pomeranian Medical University in Szczecin, Powstańców Wielkopolskich 72, 70-111 Szczecin, Poland; 2 Department of Cosmetic and Pharmaceutical Chemistry, Pomeranian Medical University in Szczecin, Powstańców Wielkopolskich 72, 70-111 Szczecin, Poland; 3 Faculty of Chemical Technology and Engineering, Department of Chemical Organic Technology and Polymeric Materials, West Pomeranian University of Technology in Szczecin, Piastów Ave. 42, 71-065 Szczecin, Poland

**Keywords:** Active pharmaceutical ingredients, Ibuprofen, Nonsteroidal anti-inflammatory drug, Cyclooxygenase, Anti-inflammatory activity, Antioxidant activity

## Abstract

Nonsteroidal anti-inflammatory drugs (NSAIDs) are widely used for pain relief and inflammation management, but there are challenges related to poor solubility and bioavailability. We explored modifications of ibuprofen (IBU) by forming ionic pairs using amino acid alkyl esters to enhance solubility without compromising the ability to inhibit cyclooxygenase (COX)-1 and COX-2). We comprehensively evaluated the pharmacological properties of the IBU derivatives, focusing on antioxidant activity (based on the ability to scavenge DPPH and ABTS), biocompatibility (using human dermal fibroblasts), and COX inhibitory potential. The antioxidant activity assays significantly enhanced DPPH scavenging activity for several IBU derivatives, particularly [l-SerOiPr][IBU], suggesting potential therapeutic benefits. There was enhanced cell viability with select derivatives, indicating possible stimulatory effects on cellular proliferation. Finally, predominant COX-1 inhibition across derivatives was consistent with IBU’s profile. This study provides insights into the pharmacological properties of IBU amino acid derivatives, highlighting their potential as therapeutic agents. Further exploration into structure–activity relationships and in vivo efficacy warranted to advance these derivatives toward clinical applications, offering prospects for novel NSAIDs with enhanced efficacy and reduced side effects.

**Supplementary Information:**

The online version contains supplementary material available at 10.1007/s43440-024-00666-6.

## Introduction

Nonsteroidal anti-inflammatory drugs (NSAIDs) are some of the most widely employed pharmaceuticals for pain relief, fever reduction, and inflammation control. It is estimated that 30–50 million people worldwide use NSAIDs daily due to their broad spectrum of activity and over-the-counter availability. Each year, more than 500 million prescriptions for NSAIDs are issued, with global production surpassing 50 million tons [1, 2]. NSAIDs are primarily carboxylic acids; they present a range of chemical structures and typically display medium-strength acidity with a p*K*_a_ range of 3–5. They are known for their analgesic, antipyretic, and anti-inflammatory effects, but their acidic nature and mechanism of action can lead to gastrointestinal and cardiovascular side effects [3–8]. To mitigate these side effects, various derivatives of parent drugs and formulations have been developed. Prodrugs, such as esters or compounds that form acidic moieties during biotransformation, are designed to pass through the stomach unchanged and activate only after enzymatic hydrolysis [9–11]. Examples include ibuprofen (IBU) amides and esters formed with heteroaromatic amines [12]; menthol, thymol, and eugenol [13]; or quercetin, salicylic alcohol, and gallic acid [14], which show improved pharmacological activity and reduced gastrointestinal side effects. Inhibition of prostaglandin E2 (PGE_2_) biosynthesis by NSAIDs has ulcerogenic effects. To avoid side effects, many preparations have been introduced in which active acid groups are obtained after enzymatic hydrolysis [15].

IBU—(*RS*)-2-[4-(2-methylpropyl)phenyl]propanoic acid—ranks as one of the most frequently employed NSAIDs for treating rheumatoid arthritis, osteoarthritis, pain, and fever. It is available over-the-counter in various forms, such as tablets, capsules, and suspensions. Rapid therapeutic action is crucial for effective pain and fever treatment, as it is directly linked to the drug's dissolution rate and absorption into the bloodstream [16]. IBU, a carboxylic acid with high lipophilicity, has low water solubility (0.076 g/dm^3^ at 25 °C), particularly in the highly acidic milieu of the stomach, necessitating higher doses that increase the risk of gastrointestinal damage. Its solubility improves significantly at higher pH, at which the molecules become ionized [16–20].

**Fig. 1:**
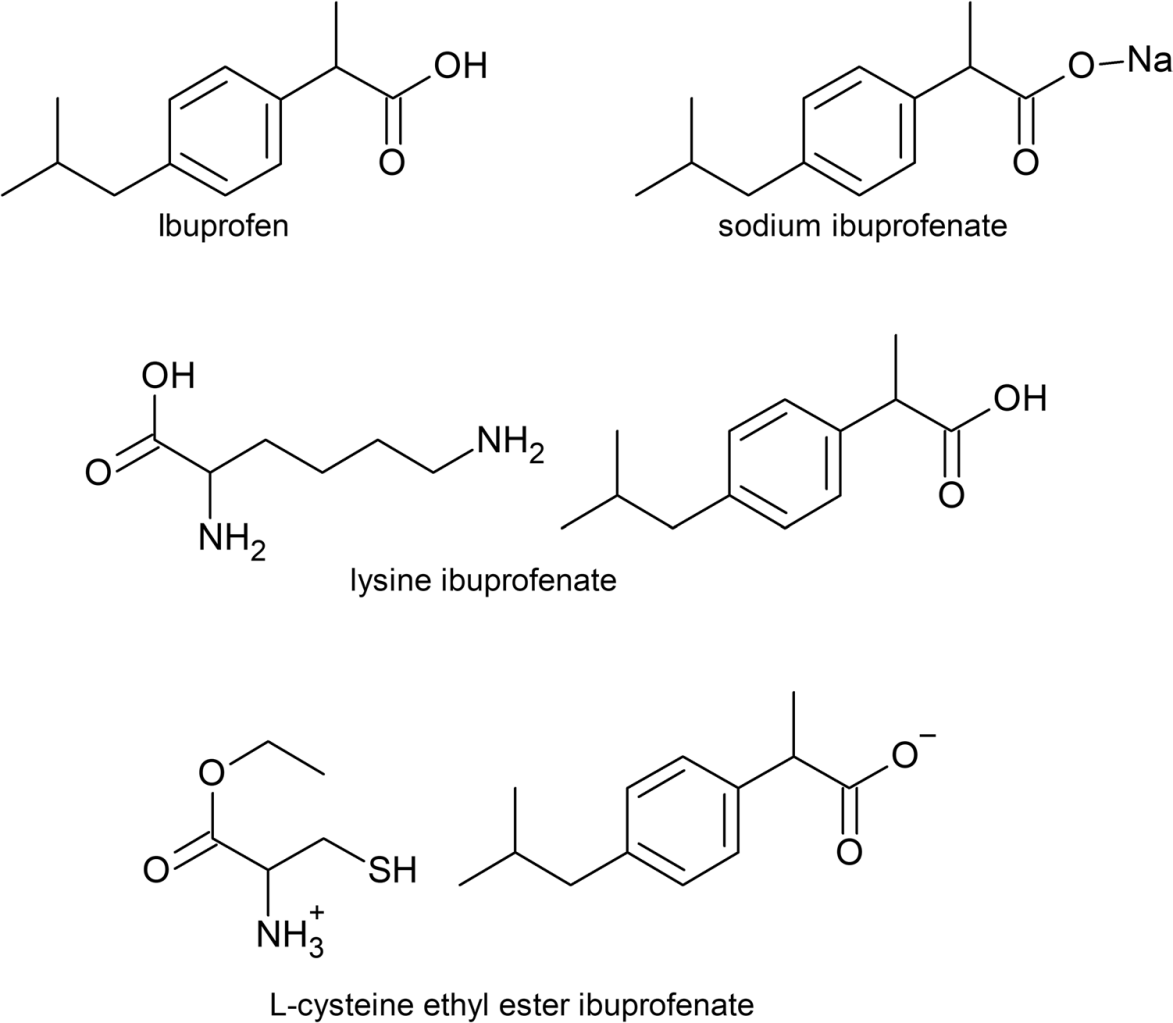
The structures of ibuprofen and its derivatives: sodium salt, lysine ibuprofenate, and L-cysteine ethyl ester ibuprofenate

Efforts to enhance the solubility and bioavailability of NSAIDs include forming their salts. Sodium, potassium, calcium, magnesium, copper, and zinc NSAID salts increase solubility in polar solvents, leading to faster therapeutic action and pain relief [21–25]. Organic cations, such as those derived from amino acids, have addressed solubility and bioavailability issues. For example, lysine ibuprofenate, a salt formed with lysine, is known for its rapid absorption and faster achievement of maximum plasma concentration than IBU acid [26]. l-Cysteine ethyl ester ibuprofenate demonstrates a robust anti-inflammatory and antioxidant capability, with fewer gastrointestinal side effects [27–30]. Other salts, such as those formed with l-proline ethyl ester, also exhibit similar advantages [31]. In addition, the IBU anion can be combined with an amino acid cation—arginine or polylysine—in which the positive charge is on the nitrogen atom of the amino group [32–35]. Figure 1 shows the structures of IBU and selected IBU derivatives.

In our previous studies, we obtained ibuprofenates of alkyl esters of various amino acids and examined the effect of modifying the length and branching of the alkyl chain and the type of amino acid on the physicochemical properties, particularly solubility, lipophilicity, and permeability through pig skin [36–46]. These salts demonstrated significantly increased water solubility, with l-lysine isopropyl ester ibuprofenate showing an increase of over 66-fold. The partition coefficient (log P) values of these salts were lower than that of IBU (log P = 3.208), with glycine isopropyl ester ibuprofenate showing the lowest log P (0.645) and l-phenylalanine isopropyl ester ibuprofenate showing the highest log P (2.207). These derivatives also exhibited excellent skin permeability, ranging from 3 times greater for the isoleucine derivative to 16 times greater for the l-lysine derivative compared with unmodified IBU. Most of the derivatives showed higher skin accumulation than IBU, except for l-aspartic acid isopropyl ester ibuprofenate, which had lower skin accumulation [36].

This work is a continuation of our existing research in which we selected the ibuprofenates of isopropyl amino acid esters for further evaluation due to their high water solubility and excellent skin permeability. In this study, we evaluated whether and how the cation in the form of an amino acid isopropyl ester affects the toxicity, binding to cyclooxygenase (COX)-1 and COX-2, and the antioxidant properties of these compounds. Many amino acids are known for their antioxidant properties, so we investigated whether these derivatives would retain this activity. We aimed to demonstrate that this approach does not reduce the interaction with COX-1 and COX-2 and, therefore, does not alter the mechanism of action or effectiveness of IBU. We present the biocompatibility, antioxidant activity, and COX inhibition results for ibuprofenate l-amino acid isopropyl esters.

## Material and methods

### Chemicals

2,2-Diphenyl-1-picrylhydrazyl (DPPH, cat. no 1898–66-4), 6-hydroxy-2,5,7,8-tetramethylchroman-2-carboxylic acid (Trolox, cat. no 53188–07-1), 2,2′-azino-bis(3-ethylbenzothiazoline-6-sulfonic acid) (ABTS, cat. no 30931–67-0), Dulbecco’s Modified Eagle Medium (DMEM) high glucose (cat. no. D1145), l-glutamine (cat. no. G7513), penicillin–streptomycin solution (cat. no. P4333), and dimethyl sulfoxide (DMSO, cat. no. D2650) were acquired from Sigma Aldrich (Saint Louis, MO, USA). Foetal bovine serum (FBS, cat. no. E5052-02) was purchased from EURx (Gdańsk, Poland). The COX Colorimetric Inhibitor Screening Assay Kit (cat. no. 701050701050) was obtained from Cayman (Ann Arbor, MI, USA). All reagents were of analytical grade.

### Synthesis of ibuprofenate l-amino acid isopropyl esters

**Fig. 2:**
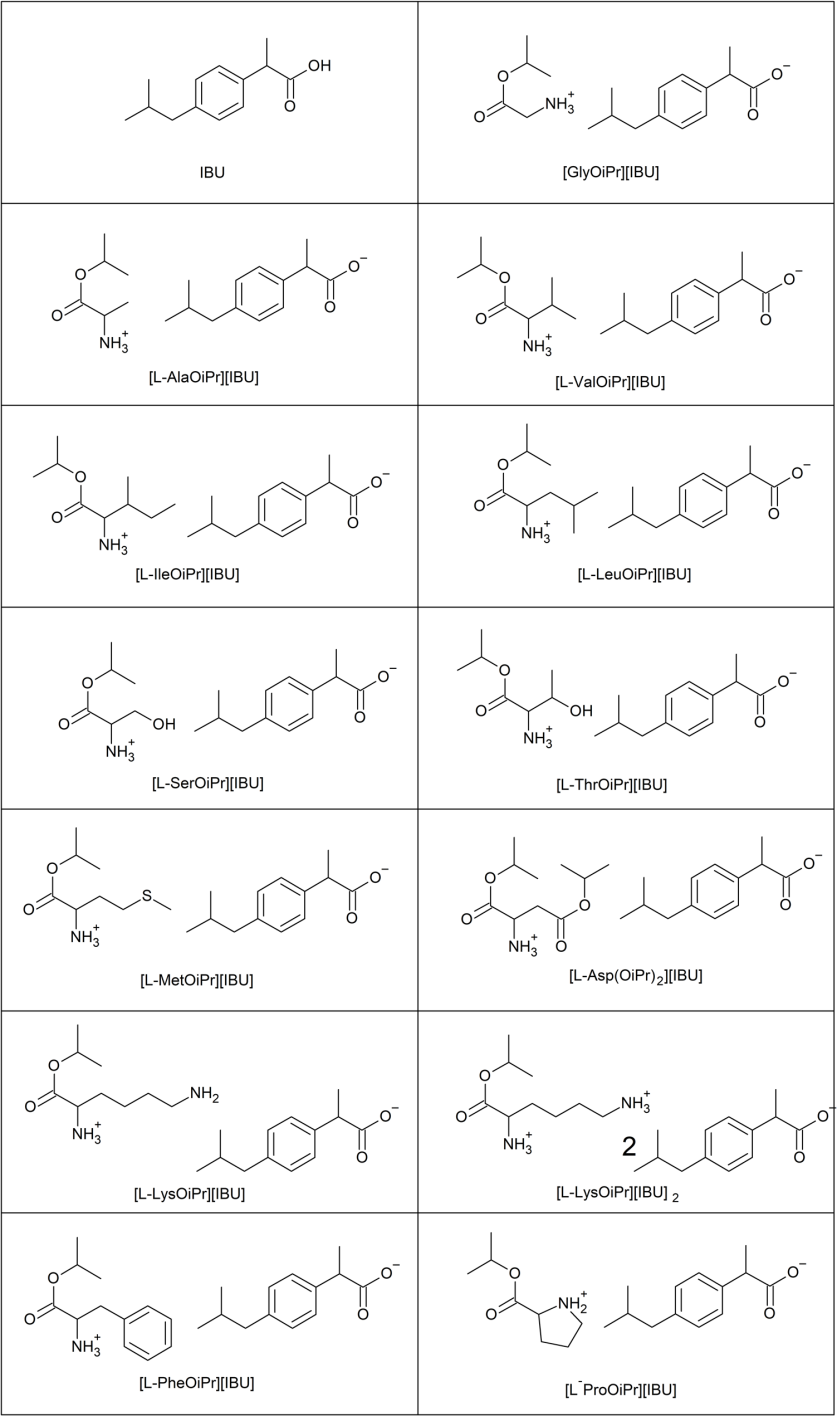
The structures and acronyms of ibuprofenates of isopropyl amino acid esters used in this study

The ibuprofenate l-amino acid isopropyl esters used in the present study were obtained according to the methodology described previously [36]. In the case of obtaining ibuprofenate isopropyl amino acid esters using racemic IBU and enantiomeric amino acid esters, two diastereomeric salts were obtained, which may have different physicochemical properties (including solubility). Therefore, the physicochemical properties were determined for the mixture of the resulting diastereoisomeric salts. The structures of the compounds used are shown in Fig. 2. The synthetic route (Figure S1, Supplementary Materials) as well as a description of the synthesis, materials used, and methods confirming the identification of the obtained compounds, are available in the Supplementary Materials. Additionally, Figures S2–S40 (Supplementary Materials) show the ^1^H and ^13^C nuclear magnetic resonance (NMR) and Fourier-transform infrared (FTIR) spectra of the obtained compounds.

### DPPH radical scavenging assay

The scavenging activity of DPPH and ABTS stable free radicals was assessed following established methods [47, 48]. Briefly, 0.15 cm^3^ of the sample solution (corresponding to 1% of the active substance) was combined with 2.85 cm^3^ of 0.3 mM DPPH radical solution prepared in 96% (v/v) ethanol. The absorbance of the DPPH working solution at 517 nm was adjusted to 1.00 ± 0.02 using 70% (v/v) ethanol. Absorbance was measured at 517 nm after incubation for 10 min in the dark at room temperature, with 70% (v/v) ethanol serving as the reference.

The ABTS stock solution was prepared by dissolving ABTS to a concentration of 7 mmol/L in a 2.45 mmol/L aqueous potassium persulfate solution. This mixture was incubated in the dark at room temperature for 24 h before being diluted with 50% (v/v) methanol to create the working solution. To perform the assay, 2.5 ml of ABTS working solution was mixed with 0.025 ml of the sample solution in a spectrophotometric cuvette. After incubation for 6 min in the dark at room temperature, the absorbance was measured at 734 nm.

The DPPH and ABTS radical scavenging activity was calculated as a percentage of inhibition using the following equation:

1 $$\%DPPH\; scavenging = 1-\frac{As}{Ac}\bullet 100\%$$

where A_s_ is the absorbance of the tested sample and A_c_ is the absorbance of the control sample.

Each sample was measured in triplicate to ensure accuracy. Trolox was used as the reference standard, and the results are expressed in mmol Trolox·g.^−3^

### Evaluation of biocompatibility

The safety study was performed on primary human dermal fibroblasts derived from the skin according to the protocol approved by the Ethics Committee at Pomeranian Medical University in Szczecin (KB-0012/02/18). The detailed protocol of fibroblast isolation, authentication, and cell culture conditions was described previously [49]. The cells were seeded in a 96-well microplate at a density of 3 × 10^3^ cells/well and cultured in DMEM high glucose supplemented with 10% heat-inactivated FBS, 2 mM l-glutamine, and penicillin–streptomycin. After 24 h, the cell culture medium was removed and replaced with 100 µl of the fresh medium containing IBU (as a reference) or its derivatives at 1000, 500, 250, 100, 10, and 1 µM. Due to their reduced solubility, the highest tested dilution for [l-LeuOiPr][IBU] and [l-LysOiPr][IBU]_2_ was 500 µM. All of the tested compounds were dissolved in dimethyl sulfoxide (DMSO, with a final concentration that did not exceed 0.2%) before use. The cells without the tested compounds (but in a medium with DMSO) were used as controls, and the highest concentrations of the tested compounds in a medium without cells were used as blanks. After 24 h, the WST-1 reagent was added to each well, and the plate was incubated for 30 min. The absorbance was measured at 450 nm (with 620 nm background correction) using a spectrophotometric microplate reader (Infinite 200 Pro, Tecan, Männedorf, Switzerland). The WST-1 assay measures the amount of tetrazolium salt reduced to formazan, which is directly proportional to the quantity of metabolically active cells. The cell viability was calculated using the formula: Atest/Acontrol × 100%. There were no differences in absorbance between wells without cells (blanks) were observed. The readings were obtained from at least three independent experiments, with each experiment conducted in triplicate. Additionally, the primary human dermal fibroblasts were imaged with an optical microscope just before the analysis using the Smart Fluorescent Cell Analyzer Microscope (JuLi, Seoul, Korea).

### COX inhibition assay

The IBU derivatives (100 µM) were tested for their ability to inhibit COX using the COX Colorimetric Inhibitor Screening Assay Kit (Cayman, cat. no. 701050701050), including both COX-1 (ovine) and COX-2 (human). Arachidonic acid (AA) was used as the substrate at a final concentration of 10 µM in the wells, and IBU (at 100 µM) served as a control inhibitor. IBU and its derivatives were incubated with COX-1/COX-2 for 5 min at 25 °C and then for 2 min at 25 °C with the colourimetric substrate (*N,N,N’,N’*-tetramethyl-phenylenediamine [TMPD]) and AA. The peroxidase activity of COX was measured colourimetrically by monitoring the appearance of oxidised TMPD at 590 nm using a spectrophotometric microplate reader (Infinite 200 Pro, Tecan, Männedorf, Switzerland). All wells contained haem as an enzyme cofactor. Wells with COX-1/COX-2 but without IBU or a derivative (potential inhibitors) were used as the 100% initial activity sample. In comparison, wells without COX and inhibitors served as background wells, with the absorbance subtracted from all other wells. The enzyme inhibition percentage was calculated using the following formula: [(100% initial activity sampl –inhibitor sample) / 100% initial activity sample] × 100%. The selectivity index was calculated as the ratio of COX-1 inhibition to COX-2 inhibition.

### In silico pharmacokinetics predictions

The SMILES (Simplified Molecular Input Line Entry System) structures of IBU and its derivatives were used for in silico pharmacokinetics predictions. Using the online SwissADME server, ADME (acronym for absorption, distribution, metabolism and excretion) attributes such as physicochemical properties, lipophilicity, water solubility, pharmacokinetics, and drug similarity properties were assessed.

### Statistical analyses

Statistical analyses were carried out using the Statistica 13 PL software (StatSoft, Kraków, Poland). The results are expressed as the mean ± standard deviation (SD). Analysis of variance (ANOVA) followed by Tukey’s test for multiple comparisons was used for statistical analysis. Comparisons were made between the IBU amino acid derivatives and the control (pure IBU) and between the IBU amino acid derivatives and pure amino acids. The antioxidant activity is also expressed as Trolox equivalents and presented as the median, minimum, and maximum values. Pearson correlation coefficients were calculated to assess the correlation between the antioxidant activity methods. For all analyses, a *p-*value < 0.05 was considered to indicate a statistically significant difference.

## Results

### Antioxidant activity

**Fig. 3:**
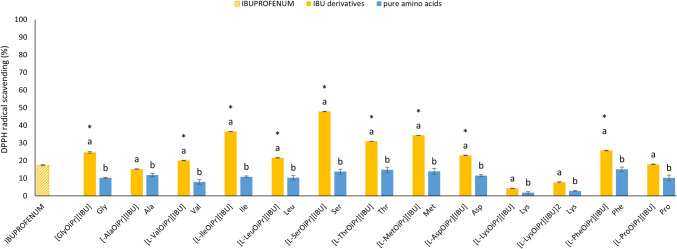
The antioxidant activity of the amino acid derivatives of ibuprofen and pure amino acid tested by the 2,2-diphenyl-1-picrylhydrazyl (DPPH) method, expressed as Trolox equivalents. All substances were analysed at a concentration of 1% (v/v, calculated as active substance). All values are presented as the mean ± standard deviation (n = 3). Statistical analysis was performed using one-way analysis of variance followed by the Tukey post hoc test. Asterisks (*) indicate a significant difference compared with the control (pure ibuprofen); different letters indicate a significant difference between the derivative and the pure amino acid, wherein a letter a denotes significantly higher antioxidant activity compared to the letter b. (α = 0.050, p < 0.0001)

**Fig. 4:**
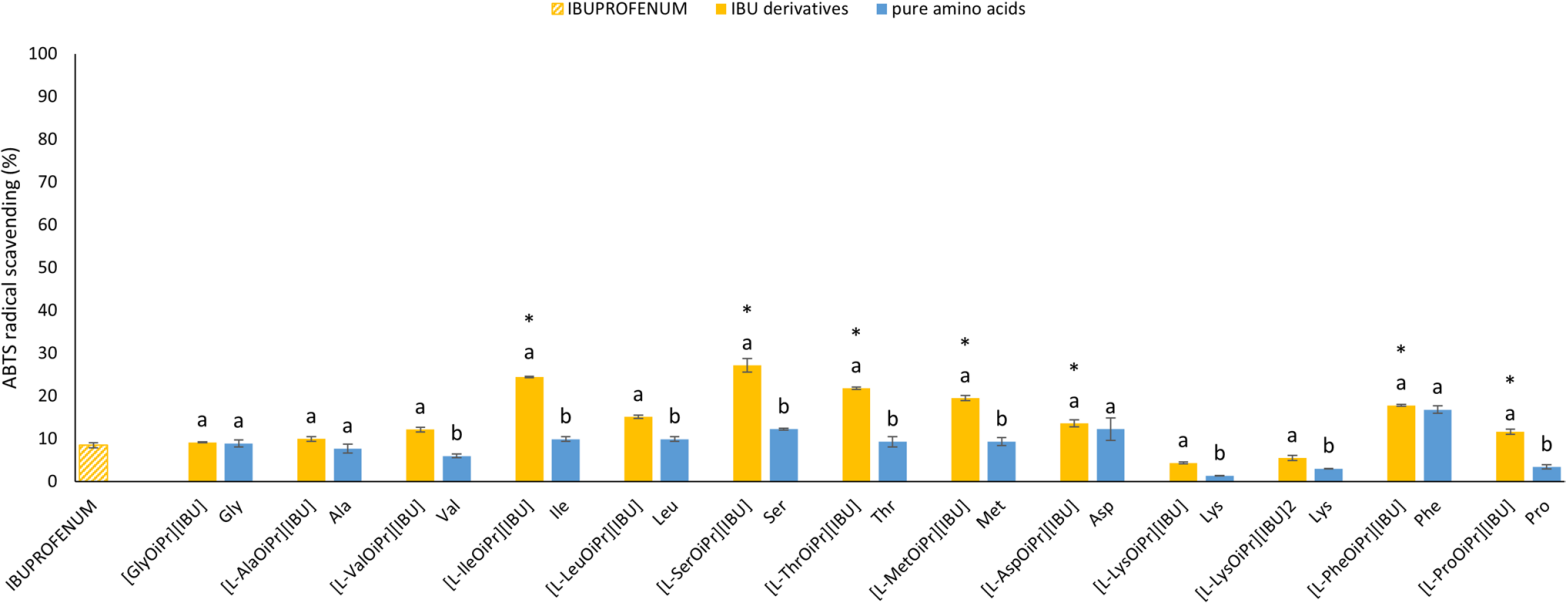
The antioxidant activity of the amino acid derivatives of ibuprofen and pure amino acid tested by the 2,2′-azino-bis(3-ethylbenzothiazoline-6-sulfonic acid) (ABTS) method, expressed as Trolox equivalents. All substances are analysed at a concentration of 1% (v/v, calculated as active substance). All values are presented as the mean ± standard deviation (n = 3). Statistical analysis was performed using one-way analysis of variance followed by the Tukey post hoc test. Asterisks (*) indicate a significant difference compared with the control (pure ibuprofen); different letters indicate a significant difference between the derivative and the pure amino acid, wherein a letter a denotes significantly higher antioxidant activity compared to the letter b. (α = 0.050, p < 0.0001)

IBU and its l-amino acid derivatives presented significant variations in their capacity to scavenge DPPH and ABTS radicals. Figure 3 shows the antioxidant activity based on the DPPH method, determining the ability of tested compounds to act as donors of hydrogen atoms or electrons to transform DPPH• into its reduced form, that is, DPPH-H [50]. Figure 4 shows the antioxidant activity based on the ABTS method.

All of the tested derivatives had the property of reducing the stable DPPH• radical. However, the ability to scavenge free radicals varied depending on the derivative. [l-SerOiPr][IBU] presented the highest DPPH scavenging activity: it was almost 2.5-fold higher compared with pure IBU. Similarly, most of the tested derivatives showed significantly higher antioxidant activity compared with the control, except for [L-ProOiPr][IBU], whose antioxidant activity did not differ significantly compared with pure IBU (F_1,4_ = 2,68, p = 0.1766). However, the following derivatives showed significantly lower DPPH radical scavenging activity compared with the control: [l-AlaOiPr][IBU] (F_1,4_ = 64.13, p = 0.00132) [l-LysOiPr][IBU] (F_1,4_ = 2191.8, p = 0.00000), and [l-LysOiPr][IBU]_2_ (F_1,4_ = 876.01, p = 0.00001). The following derivatives showed significantly higher ABTS radical scavenging activity compared with pure IBU: [l-LeuOiPr][IBU] (F_1,4_ = 679.69, p = 0.00001) [l-SerOiPr][IBU] (F_1,4_ = 383.14, p = 0.00004,), [l-ThrOiPr][IBU] (F_1,4_ = 6432.0, p = 0.00000), [l-MetOiPr][IBU] (F_1,4_ = 927.45, p = 0.00001), [l-AspOiPr] [IBU] (F_1,4_ = 81.147, p = 0.00084), [l-PheOiPr][IBU] (F_1,4_ = 927.45, p = 0.00001), and [l-ProOiPr][IBU] (F_1,4_ = 52.839, p = 0.00190). Most of the analysed IBU amino acid derivatives showed significantly higher antioxidant activity compared with the control (pure IBU), except for the l-Ala and l-Lys derivatives. These findings suggest a relationship between antioxidant activity and the amino acid used for synthesis. The lysine derivatives presented the lowest antioxidant activity (2.803% DPPH inhibition and 3.033% ABTS inhibition), while the phenylalanine derivatives had the highest activity (15.167% DPPH inhibition and 16.815% ABTS inhibition). There were also significant differences between the derivatives and the pure amino acids. For the DPPH method, this applied to all derivatives, while for the ABTS method, it applied to all derivatives except [GlyOiPr][IBU] (F_1,4_ = 3.2961, p = 0.14362), [l-AlaOiPr][IBU] (F_1,4_ = 1.8461, p = 0.24581), [l-AspOiPr][IBU] (F_1,4_ = 0.73051, p = 0.44089), and [l-PheOiPr][IBU] (F_1,4_ = 3.7584, p = 0.1245).

The antioxidant activity analysis considering Trolox equivalents is presented in Tables S1 and S2 (Supplementary Materials). For the DPPH method, the derivatives presented antioxidant activity ranging from 0.060 ± 0.015 mmol Trolox/dm^−3^ for [l-LysOiPr][IBU]_2_ to 0.413 ± 0.012 mmol Trolox/dm^−3^ for [l-SerOiPr][IBU]. For this method, the derivatives showed significantly higher activity compared to the control (pure IBU: 0.145 ± 0.018 mmol Trolox/dm^−3^). For the ABTS method, the antioxidant activity ranged from 0.073 ± 0.011 mmol Trolox/dm^−3^ for [l-LysOiPr][IBU]_2_ to 1.109 ± 0.072 mmol Trolox/dm^−3^ for [l-SerOiPr][IBU]. The vast majority of the derivatives also showed a significant difference compared with the pure amino acids (Table S1, Supplementary Materials). Figure S41 (Supplementary Materials) shows the Pearson correlation coefficients for the DPPH and ABTS antioxidant activity. There was a strong and significant relationship between these parameters for IBU derivatives (r = 0.9517) and pure amino acids (r = 0.7382) (Figure S42, Supplementary Materials).

### Evaluation of biocompatibility

**Fig. 5:**
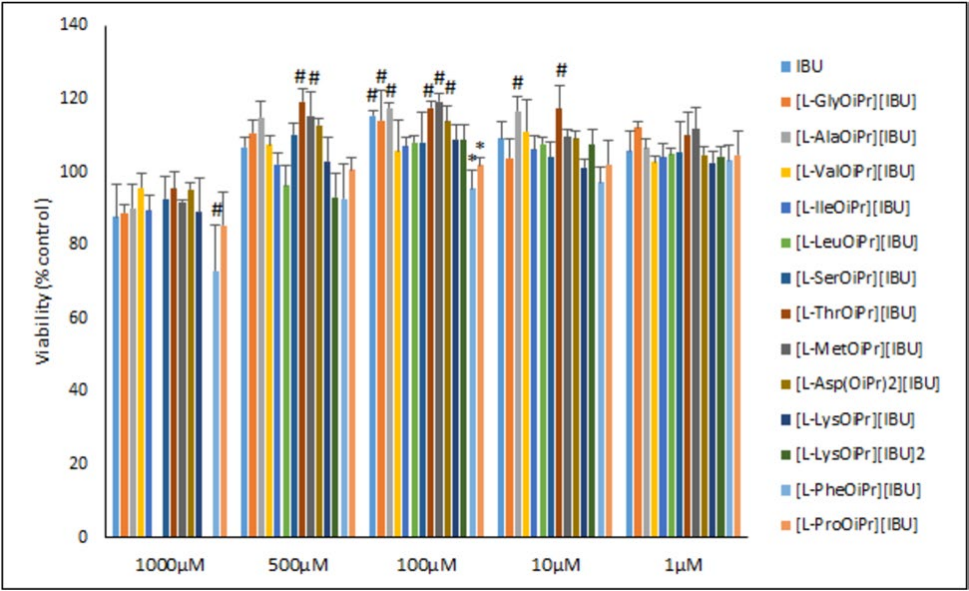
The viability of primary human dermal fibroblasts determined with the WST-1 assay after treatment for 24 h with the ibuprofen amino acid derivatives and pure ibuprofen at the indicated concentrations. All values are presented as the mean ± standard deviation from at least three independent experiments. Statistical analysis was performed using analysis of variance followed by the Tukey post hoc test. An asterisks (*) indicate a significant difference compared with the control (pure ibuprofen). A pound sign (^#^) indicates a significant difference compared with the control (non-treated cells)

We evaluated the biocompatibility of the IBU amino acid derivatives in primary human dermal fibroblasts in relation to the cell metabolic activity (WST-1) assay. Compared with the control, there was a significant reduction in cell viability (to 72.8% ± 12.8%) only with 1000 µM [l-PheOiPr][IBU] (ANOVA F_12,28_ = 2.325341, p = 0.032320, Tukey post hoc test p = 0.008237). For the remaining concentrations and derivatives, there was a notable difference compared with the control due to an increase in cell viability. The differences occurred most frequently at 100 µM (ANOVA F_14,32_ = 7.623800, p = 0.000001) (Fig. 5), a concentration at which pure IBU increased cell viability to 115.3% ± 1.5% (p = 0.014446, Tukey post hoc test). Moreover [l-GlyOiPr][IBU], [l-AlaOiPr][IBU], [l-ThrOiPr][IBU], [l-MetOiPr][IBU], [l-AspOiPr][IBU] increased cell viability to 114.13 ± 8.25% (p = 0.032084, Tukey post hoc test), 117.49 ± 1.30% (p = 0.003185, Tukey post hoc test), 117.40 ± 1.70% (p = 0.003383, Tukey post hoc test), 119.22 ± 2.32% (p = 0.000960, Tukey post hoc test) and 113.74 ± 4.32% (p = 0.041091, Tukey post hoc test), respectively It should be emphasised that [l-ThrOiPr][IBU] significantly promoted cell proliferation at 500 µM (ANOVA F_14,32_ = 8.511934, p = 3.20905990402842E-07, followed by Tukey post hoc test p = 0.003718), 100 µM (ANOVA F_14,32_ = 7.623800, p = 0.000001, followed by Tukey post hoc test p = 0.003383) and 10 µM ((ANOVA F_14,32_ = 4.928118, p = 0.000093, followed by Tukey post hoc test p = 0,004954). We observed significant differences between IBU and its derivatives only in the case of 100 µM [l-PheOiPr][IBU] and [l-ProOiPr][IBU], with cell viability of 95.5% ± 5.0% (ANOVA F_14,32_ = 7.623800, p = 0.000001, followed by Tukey post hoc test p = 0.000368) and 101.9% ± 2.2% ANOVA F_14,32_ = 7.623800, p = 0.000001, followed by Tukey post hoc test p = 0.033370), respectively. There were no differences between IBU, its derivatives, and non-treated cells at 1 µM. Figure S42 (Supplementary Materials) shows images of cells treated with the highest concentration of the tested compounds. All cells exhibited the typical spindle shape of human dermal fibroblasts, and the cultures appeared to be uniformly confluent, with no signs of cytotoxicity, after treatment for 24 h.

### COX-1 and COX-2 inhibition

**Fig. 6:**
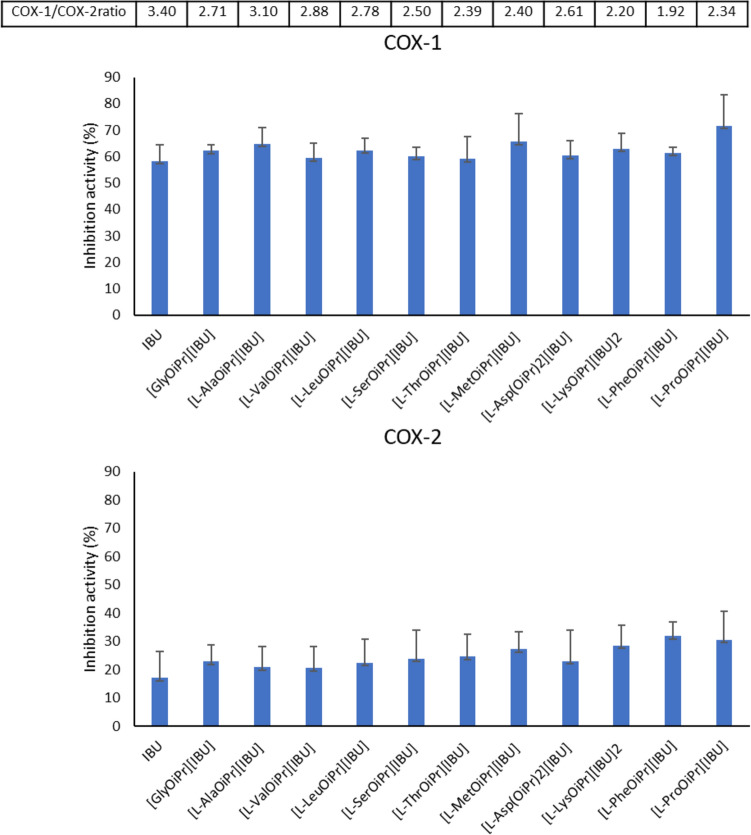
The in vitro inhibitory activity of the ibuprofen amino acid derivatives and pure ibuprofen against cyclooxygenase (COX)-1 and COX-2 determined using the COX Colorimetric Inhibitor Screening Assay Kit. All substances were analysed at 100 µM. All values are presented as the mean ± standard deviation from at least three independent experiments. Analysis of variance was used for statistical analysis; there were no significant differences

We evaluated the ability of IBU and its amino acid derivatives to inhibit COX-1 and COX-2. This data provides crucial insights into the pharmacological profile of IBU derivatives, particularly in terms of their selectivity against these enzymes. IBU is classified as a non-selective NSAID but with some preference towards COX-1 [51, 52]. The COX Colorimetric Inhibitor Screening Assay confirmed that both the reference IBU and its derivatives showed 2–3 times greater inhibition of COX-1 relative to COX-2 (Fig. 6). IBU inhibited COX-1 and COX-2 activity by 58.3% ± 6.2% and 15.2% ± 9.5%, respectively. Notably, there were no significant differences between the tested derivatives and the reference drug with regard to the inhibition of either enzyme and the COX-1/COX-2 ratio (the selectivity index).

### In silico pharmacokinetics predictions

Based on pharmacokinetic analysis of the IBU derivatives performed using the SwissADME tool (Table S3, Supplementary Materials), these compounds are predicted to have increased solubility (except for [L-LysOiPr][IBU]_2_ and [L-PheOiPr][IBU]) and high absorption in the gastrointestinal tract, except for [L-LysOiPr][IBU]_2_. The moderate solubility of [L-LysOiPr][IBU]_2_ could be responsible for the expected lower gastrointestinal absorption. On the other hand, similarly to IBU, [GlyOiPr][IBU], [l-AlaOiPr][IBU], [l-SerOiPr][IBU], [l-Asp(OiPr)_2_][IBU], and [l-ProOiPr][IBU] are not substrates for P-glycoprotein, which may have a beneficial effect on their bioavailability. All derivatives comply with Lipinski’s rules (except [L-LysOiPr][IBU]_2_, which has a molecular weight > 500 kDa), suggesting their potential good oral bioavailability. The lack of detected PAINS alerts and the low synthetic complexity indicate that these compounds have a low potential to generate false positive results in bioanalysis and are possible to produce on an industrial scale. Taken together, the IBU derivatives present a promising pharmacokinetic profile, which may be beneficial in further preclinical and clinical studies.

## Discussion

We explored the pharmacological properties of IBU derivatives by forming ion pairs with amino acid alkyl esters. Our main findings revealed that these modifications enhance the antioxidant properties of IBU, improve biocompatibility, and maintain inhibitory activity against COX-1 and COX-2. Specifically, [l-SerOiPr][IBU] demonstrated superior DPPH and ABTS radical scavenging activity. These results suggest that IBU l-amino acid derivatives could be promising candidates for developing NSAIDs with improved therapeutic benefits and fewer side effects.

### Antioxidant activity

The results regarding the antioxidant activity of IBU and its l-amino acid derivatives provide significant insights into their potential therapeutic effects. Oxidative stress results from an imbalance between reactive oxygen species (ROS) and antioxidant levels, leading to cellular damage and health-threatening processes. ROS typically include oxygen-based radicals such as the hydroxyl radical and the superoxide anion radical, as well as non-radical species such as hydrogen peroxide and singlet oxygen. They contribute to the development of several conditions, including, among others, atherosclerosis, inflammatory injuries, cardiovascular diseases, cancer, neurodegeneration, and ageing [37, 53]. Some studies have indicated that the antioxidant properties of NSAIDs may play a role in enhancing their anti-inflammatory effects [54, 55].

The findings from the antioxidant activity assays highlight the potential therapeutic benefits of the IBU derivatives. The significantly enhanced DPPH scavenging activity of most derivatives, especially [l-SerOiPr][IBU], suggests that these modifications can enhance the antioxidant properties of IBU. For the DPPH method, the antioxidant activity of the majority of the tested derivatives was significantly higher compared with the control, except for [l-AlaOiPr][IBU], [l-LysOiPr][IBU], [l-LysOiPr][IBU]_2_, and [l-ProOiPr][IBU]. On the other hand, the ABTS method revealed significantly higher antioxidant activity for [l-LeuOiPr][IBU], [l-SerOiPr][IBU], [l-ThrOiPr][IBU], [l-MetOiPr][IBU], [l-AspOiPr] [IBU], [l-PheOiPr][IBU], and [l-ProOiPr][IBU]. The variability in activity based on the amino acid derivative indicates a complex relationship between the structure of the amino acid and its antioxidant capability. The relatively low antioxidant activity of the amino acids alone, coupled with the higher activity of their IBU derivatives, suggests a synergistic effect that merits further investigation.

There was no direct correlation between the type of amino acid and antioxidant activity. Some derivatives demonstrate much higher DPPH and ABTS inhibition than the total value for individual compounds would indicate. Synergism can be proposed, and an appropriate structure of IBU derivatives can positively affect the result.

### Evaluation of biocompatibility

The biocompatibility results obtained in our study are promising. Namely, most of the IBU derivatives not only maintained cell viability but also enhanced it at certain concentrations. The significant increase in cell viability, observed with several derivatives, indicates their potential to promote cell proliferation, which could be beneficial in tissue repair and regenerative medicine. The lack of cytotoxic effects at high concentrations further underscores the safety profile of these derivatives. These findings align with previous studies demonstrating the stimulatory effects of COX inhibitors on various cell types, suggesting the broader applicability of these derivatives in therapeutic contexts [56–59]. Our previously published data also revealed that low concentrations of naproxen-based amino acid ester salts could stimulate the proliferation of RAW 264.7 cells, a murine macrophage cell line [56]. Among these, the most active compound was [l-ValOiPr][NAP], which increased cell viability to nearly 150% and 160% at concentrations of 1 and 10 µM, respectively. It is important to note that the reference naproxen (a non-selective NSAID) also increased cell viability at 1 and 10 µM, reaching 130% and 120%, respectively. Similarly to our data, Krzyżak et al. [57] revealed that some novel *N*-substituted 1H-pyrrolo[3–c]pyridine-1,3(2H)-diones COX inhibitors stimulated normal human dermal fibroblast proliferation at 10 and 50 µM. In addition, there was a proliferation of lung fibroblasts in the presence of 5 μM indomethacin, a non-selective COX inhibitor. According to the authors, the results proved that fibroblast-derived prostaglandin(s) PG(s), likely PGE_2_, suppresses cell proliferation [58]. Fibroblasts are not the only cells that can be stimulated by COX inhibitors. Non-selective COX inhibitors have been reported to stimulate the proliferation of hematopoietic stem cells, enhance bone marrow and splenic hematopoiesis, and promote bone marrow erythropoiesis in mice [59].

### COX-1 and COX-2 inhibition

As expected, the IBU amino acid derivatives did not change the pharmacophoric pattern of the initial structure and did not affect their pharmacodynamic properties. The COX inhibition assays revealed that the IBU amino acid derivatives maintained a preference for COX-1 inhibition, consistent with IBU’s known pharmacological profile. The confirmation of the preservation of the pharmacodynamic activity of the IBU demonstrates the validity of the modifications made, as incorrect formulation could have a negative impact on the overall therapeutic and pharmaceutical activity of the active pharmaceutical ingredients [60].

### Structure–activity relationship analysis

Our study highlights the intricate relationship between the structure of the IBU amino acid derivatives and their pharmacological properties. The presence of amino acid esters can significantly enhance the antioxidant properties and biocompatibility of IBU, while maintaining its COX inhibition profile. The variability observed in the antioxidant activity, and biocompatibility among the derivatives underscores the importance of the specific amino acid used in the ester formation.

The enhanced antioxidant activity observed for derivatives such as [l-SerOiPr][IBU] and [l-PheOiPr][IBU] can be attributed to the presence of functional groups that stabilise free radicals. Meanwhile, the ability of several derivatives, including [l-GlyOiPr][IBU], [l-AlaOiPr][IBU], [l-ThrOiPr][IBU], [l-MetOiPr][IBU], and [l-AspOiPr][IBU], to improve fibroblast viability suggests that these amino acids may confer additional benefits related to cellular health and proliferation. It should be emphasized that the better antioxidant and pro-proliferative properties of the IBU amino acid derivatives, which constitute an undoubted advantage when administering the drug dermally, may not be as pronounced when administered systemically because of the low physical stability of ion-pair formulations. It is well documented that when ion pairs distribute in the body, they almost certainly dissociate into their parent ions [61, 62].

The in silico ADME predictions revealed that most of the IBU amino acid derivatives demonstrate improved solubility, a finding that is supported by our previously published data [36]. Additionally, we observed a certain relationship between the pharmacokinetic properties of ibuprofenate amino acid isopropyl esters and their biological activity. Specifically, the derivative with moderate solubility ([L-PheOiPr][IBU]) was the only one among the tested derivatives that significantly reduced cell viability compared with the control. On the other hand, we could not test ([L-LysOiPr][IBU]_2_), classified as ‘moderately soluble’ at all concentrations during the in vitro biocompatibility study. Furthermore, [L-LysOiPr][IBU]_2_ was the only derivative that did not meet the criteria of Lipinski's rule of five. In general, derivatives with higher solubility demonstrated enhanced antioxidant activity, likely due to better interaction with the aqueous environments where free radicals are present.

## Conclusions

Our study revealed diverse antioxidant activity among IBU amino acid derivatives, with some exhibiting superior DPPH and ABTS radical scavenging compared with pure IBU. For both methods, [l-SerOr][IBU] had the highest antioxidant activity compared with pure IBU. Importantly, the choice of amino acid for synthesis appears to influence antioxidant activity, suggesting a discernible structure–activity relationship. Furthermore, certain derivatives displayed synergistic effects, with enhanced antioxidant efficacy beyond the sum of the individual components.

Evaluation of biocompatibility demonstrated that the IBU amino acid derivatives generally enhanced the viability of primary human dermal fibroblasts, indicating potential stimulatory effects on cellular proliferation. Notably, some of the derivatives significantly increased cell viability compared with the control, underscoring their biocompatibility and potential for tissue repair and regeneration. Importantly, the absence of cytotoxic effects at high concentrations further supports the safety profile of the IBU derivatives.

Analysis of COX inhibition revealed that IBU and its derivatives predominantly exhibited a preference towards COX-1 inhibition over COX-2, aligning with the classification of IBU as a non-selective NSAID. While overall inhibition patterns were similar, we observed subtle differences among the derivatives, suggesting the potential for fine-tuning selectivity. This ability could be useful to modulate inflammation.

The formation of ion pairs between IBU and amino acid alkyl esters significantly impacts the pharmacological properties of the resulting derivatives. Our findings suggest that the choice of amino acid ester is crucial in determining the antioxidant activity, biocompatibility, and COX inhibition profile of IBU derivatives. This study provides a basis for further exploration into the structure–activity relationships of NSAID derivatives, potentially leading to the development of novel therapeutic agents with enhanced efficacy and reduced side effects. Further research should focus on in vivo studies to confirm the therapeutic benefits and safety profiles of these derivatives. Additionally, exploring a broader range of amino acid esters could provide deeper insights into the relationship between molecular structure and pharmacological activity, paving the way for the design of more effective and safer NSAIDs.

## Supplementary Information

Below is the link to the electronic supplementary material.

Supplementary file1 (PDF 3578 KB)

## Data Availability

The datasets generated during or analyzed during the current study are available from the corresponding author upon reasonable request.
