# Supplementary material for: Comprehensive evaluation of ibuprofenate amino acid isopropyl esters: insights into antioxidant activity, cytocompatibility, and cyclooxygenase inhibitory potential
Source: Pharmacol Rep. 2024 Oct 19;76(6):1470–81. doi: 10.1007/s43440-024-00666-6 (PMC11582120; doi:10.1007/s43440-024-00666-6)
Supplement: Supplementary file 1 — Supplementary file1 (PDF 3578 KB) [file 43440_2024_666_MOESM1_ESM.pdf]

Supplementary Materials

Comprehensive Evaluation of Ibuprofenate Amino Acid Isopropyl Esters: Insights into Antioxidant Activity, Cytocompatibility, and COX Inhibitory Potential

Magdalena Perużyńska[1], Anna Nowak[2], Anna Muzykiewicz-Szymańska[2], Łukasz Kucharski[2], Joanna Klebeko[3], Karolina Bilska[3], Ewelina Kopciuch[3], Radosław Birger [1], Marek Droździk [1], Paula Ossowicz-Rupniewska[3]

[1] Department of Experimental and Clinical Pharmacology, Pomeranian Medical University in Szczecin, Powstańców Wlkp. 72, 70-111 Szczecin, Poland

[2] Department of Cosmetic and Pharmaceutical Chemistry, Pomeranian Medical University in Szczecin, Powstańców Wielkopolskich Ave. 72, 70-111 Szczecin, Poland

[3] Department of Chemical Organic Technology and Polymeric Materials, Faculty of Chemical Technology and Engineering, West Pomeranian University of Technology in Szczecin, Piastów Ave. 42, 71-065 Szczecin, Poland

Materials

Glycine (purity ≥99%), L-alanine (purity ≥98.5%), L-valine (purity ≥98.5%), L-leucine (purity ≥98.5%), L-serine (purity ≥98.5%), L-methionine (purity ≥99%), L-phenylalanine (purity ≥98.5%), L-proline (purity ≥98.5%) were purchased from ROTH. L-isoleucine (purity ≥98%), L-threonine (purity ≥97%), and L-aspartic acid (purity ≥98%) were purchased from Fluorochem. L-lysine (purity ≥97%), (*R*,*S*)-2-(4-isobutylphenyl)propanoic acid (ibuprofen, racemic mixture, purity ≥98%) were provided from AmBeed. Chlorotrimethylsilane (purity ≥98%) and DMSO-d_6 (99.9 atom% D) were purchased from Sigma-Aldrich. $CDCl_3$ (purity 99.8%; containing 0.03% TMS) was purchased from Eurisotop. Propan-2-ol (ACS), chloroform (ACS) and ammonia solution (25%) were provided from Stanlab. Na_2SO_4 (ACS) and NaCl (ACS) were purchased from Chempur.

Methods

Nuclear magnetic resonance spectroscopy (NMR)

^{1}H NMR and ^{13}C NMR were recorded on a BRUKER DPX-400 spectrometer (400 MHz and 100 MHz). All NMR chemical shifts were reported with the solvent resonance as an internal standard.

For ^{1}H NMR: $CDCl_3$ = δ 7.26 ppm, DMSO-d_6= δ 2.50 ppm. For ^{13}C NMR: $CDCl_3$ = δ 77.1 ppm, DMSO-d_6 = δ 39.8 ppm

Total reflectance – Fourier transform infrared spectroscopy (ATR-FTIR)

ATR-FTIR spectra data were recorded on Thermo Scientific Nicolet 380 spectrometer equipped with an ATR diamond plate. The spectra were recorded in transmission mode in the range of 4000 – 400 cm^{-1} at 4 cm^{-1}.

Synthesis of amino acids isopropyl ester ibuprofenates

The general scheme of the reaction is presented in Figure S2. In the first step, about 5 g of amino acid was dispersed into 50 mL of alkyl alcohol at room temperature. Then, two molar equivalents of TMSCl were added to the mixture. The solution was stirred thoroughly at 60ºC for complete conversion, as manifested by the dissolution of substrates. Then, the excess of TMSCl and alcohol and formed by-products (TMSOH or TMSR) were removed by evaporation at 60°C under a vacuum. The product was purified from the residue by washing with diethyl ether. The obtained hydrochloride was dried in a vacuum dryer at 60°C, 5 mbar for 24 h. As a result, the L-valine alkyl ester hydrochloride (ValOR·HCl) was obtained with a good yield (94-99%). The next step obtained in the first step [AAOiPr][HCl] was added to a small amount of distilled water and neutralized by adding one to three molar equivalents of 25% ammonium hydroxide aqueous solution. The solution was intensively mixed, and then the product was extracted with diethyl ether. The organic layer was dried using anhydrous Na_2SO_4 and then concentrated under a vacuum to receive [AAOiPr]. The last step is the protonation of the amino group of the amino acid with acid, which results in the final product. First, the appropriate amino acid isopropyl ester (1.0 mmol) was added to ibuprofen (1.0 mmol), and it was mixed for 30 minutes at room temperature. After the reaction had ended, the product was dried for 24 h at 50 °C under reduced pressure. All syntheses were performed thrice on a 0.5 - 5 g product scale. The purity and identity of obtained compounds were confirmed by NMR and FT-IR.

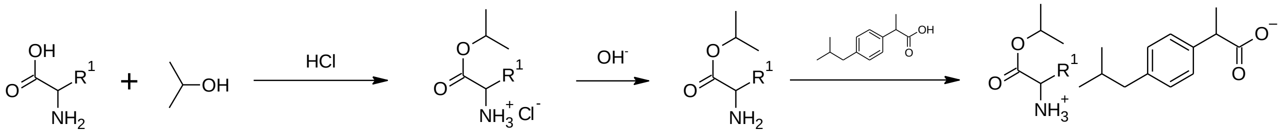

Figure S1. General synthesis path of [AAOiPr][IBU].

The NMR spectra of [AAOiPr][IBU]

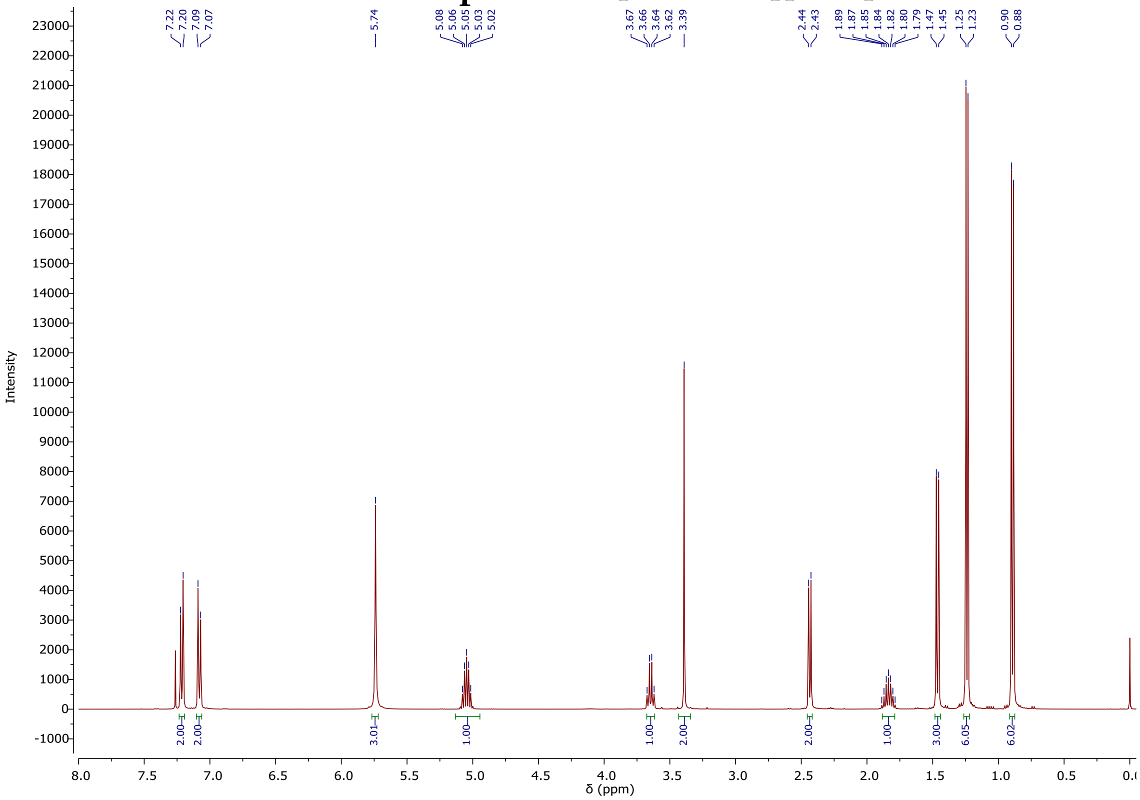

Figure S2. ^{1}H NMR spectra of [GlyOiPr][IBU].

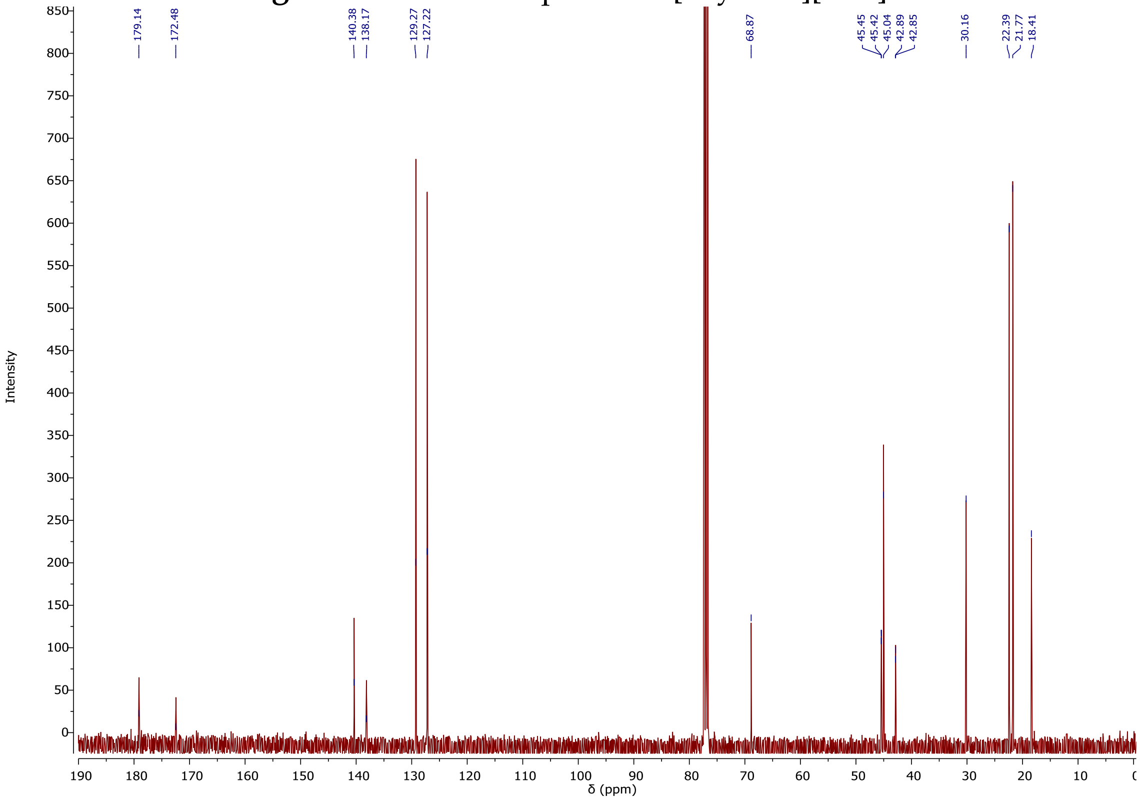

Figure S3. ^{13}C NMR spectra of [GlyOiPr][IBU].

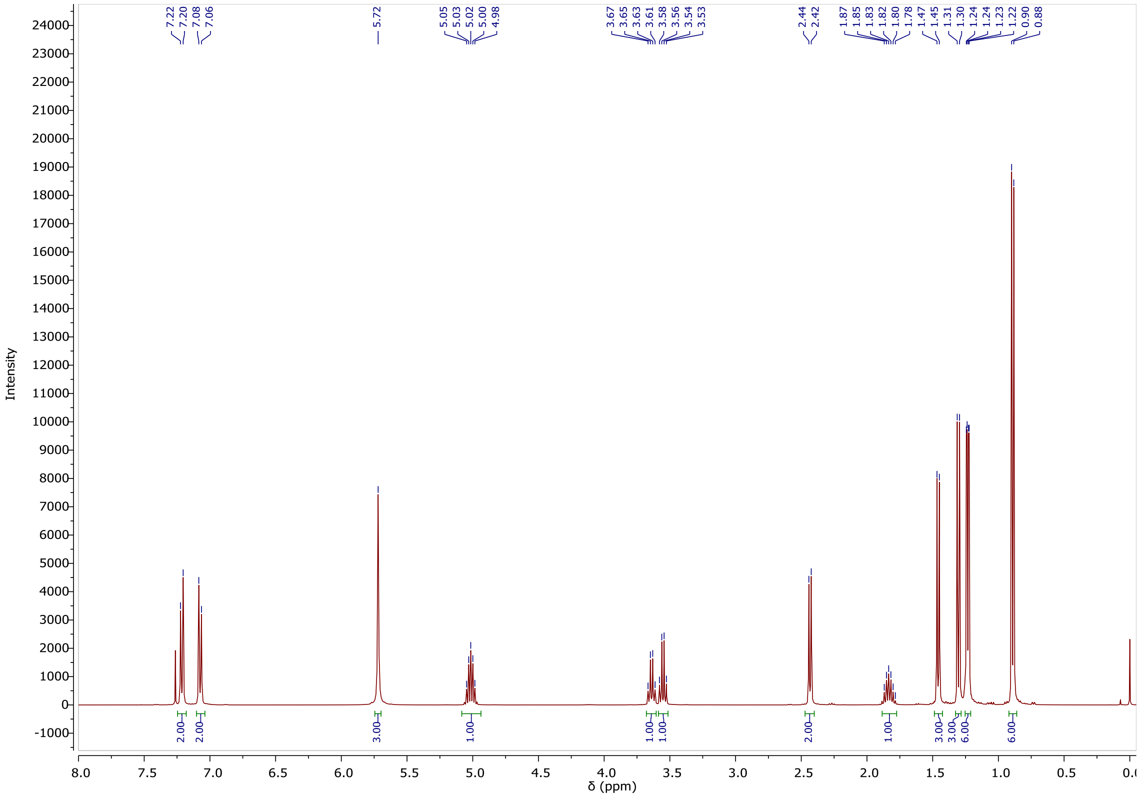

Figure S4. ^{1}H NMR spectra of [L-AlaOiPr][IBU].

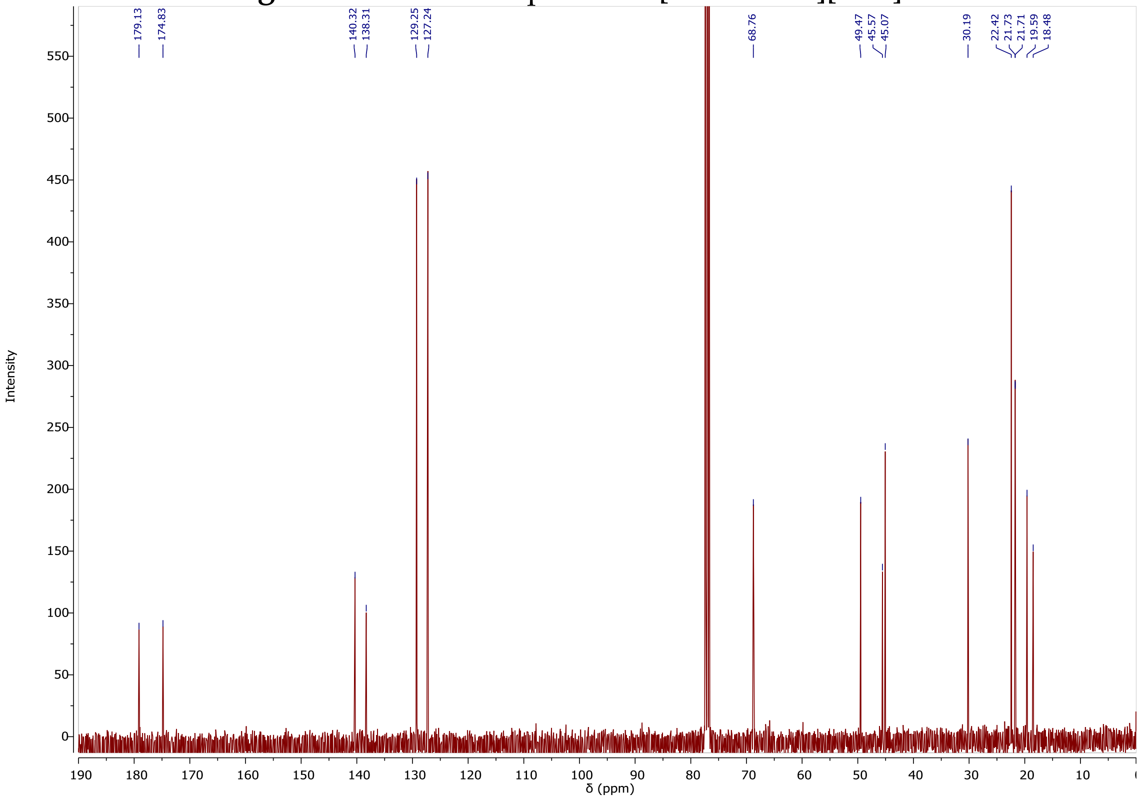

Figure S5. ^{13}C NMR spectra of [L-AlaOiPr][IBU].

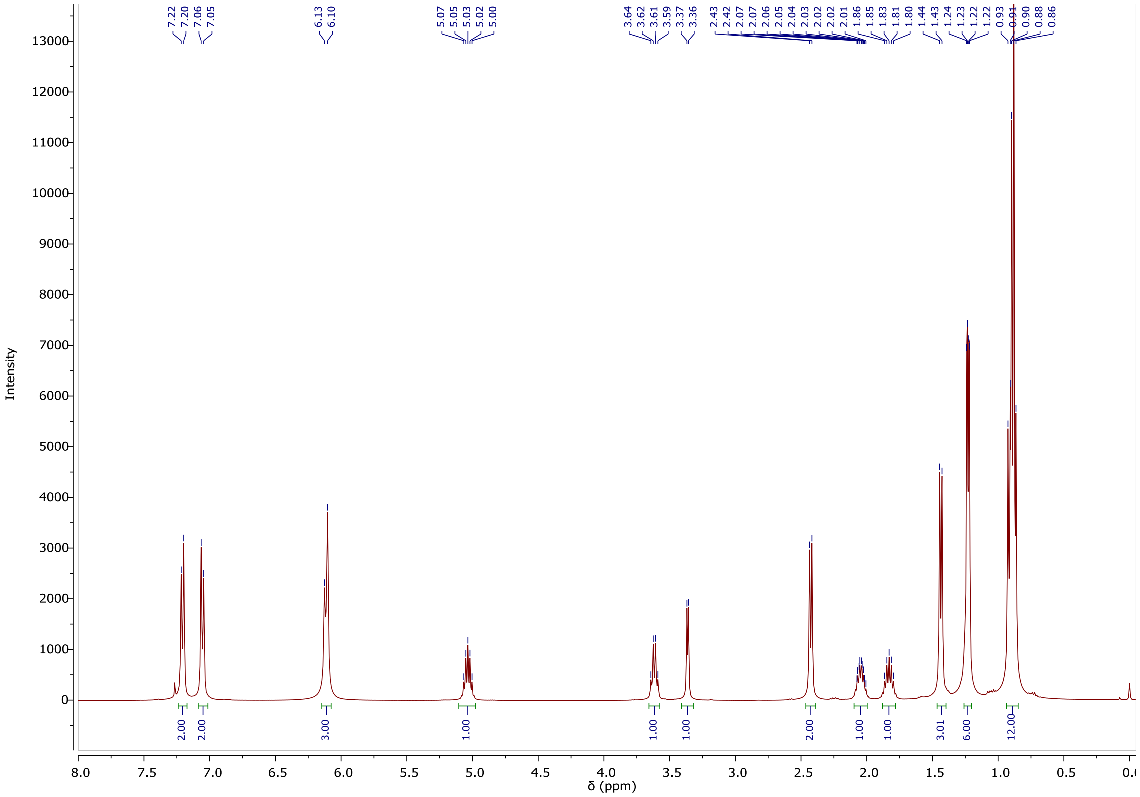

Figure S6. ^{1}H NMR spectra of [L-ValOiPr][IBU].

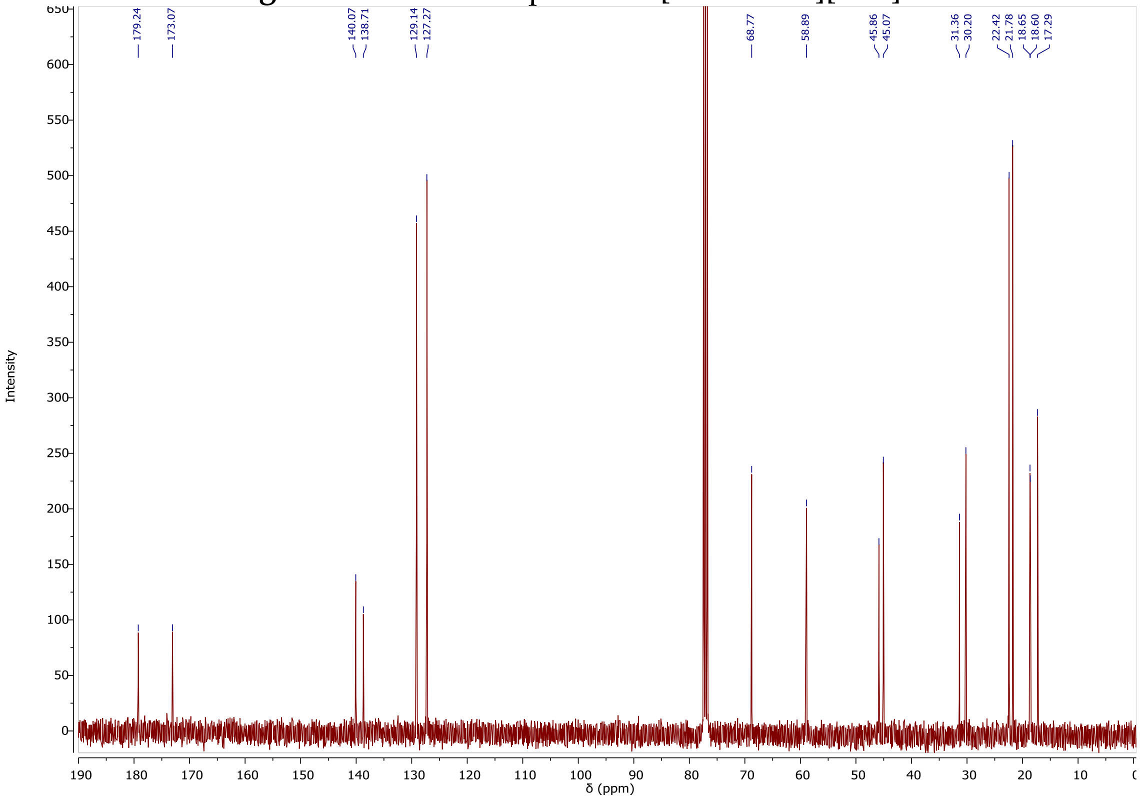

Figure S7. ^{13}C NMR spectra of [L-ValOiPr][IBU].

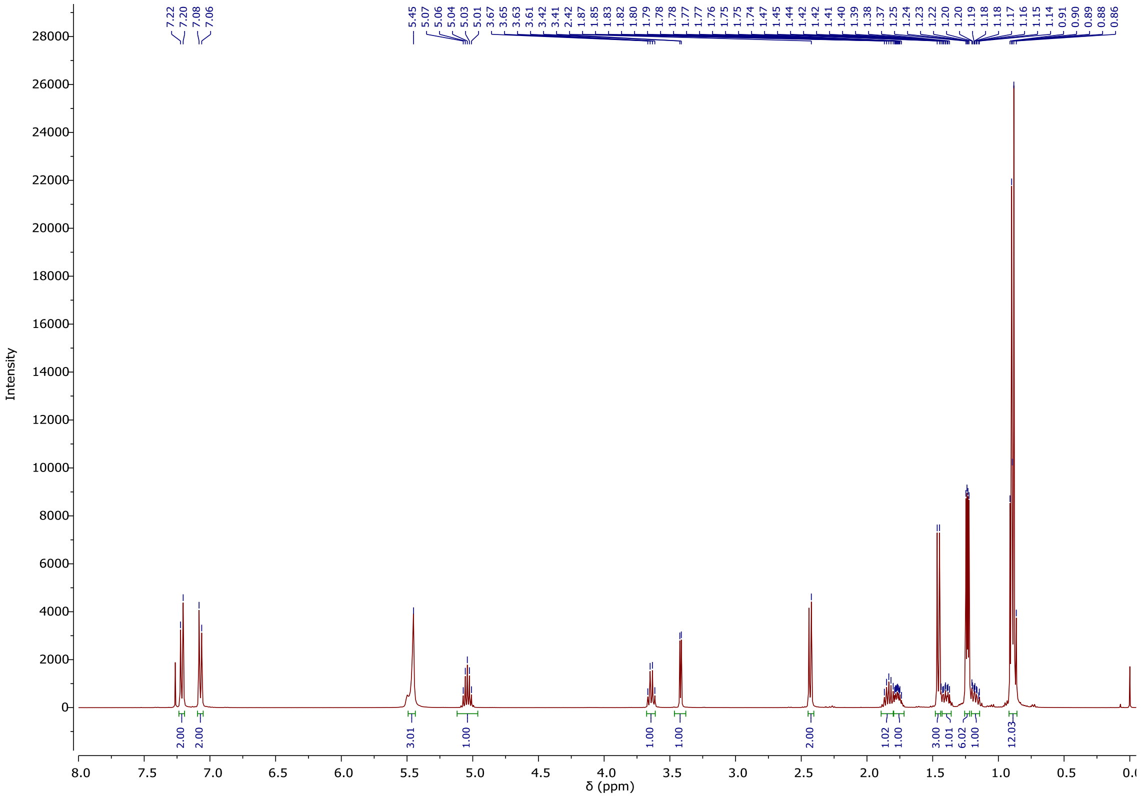

Figure S8. ^{1}H NMR spectra of [L-IleOiPr][IBU].

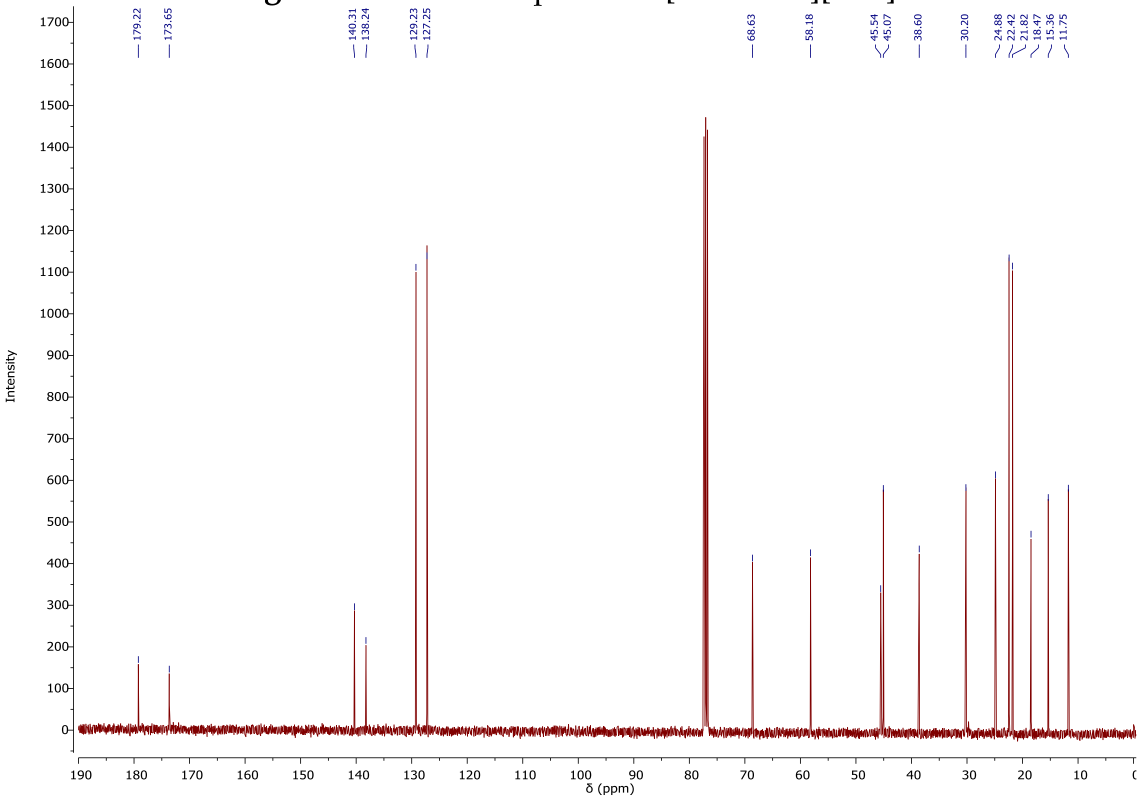

Figure S9. ^{13}C NMR spectra of [L-IleOiPr][IBU].

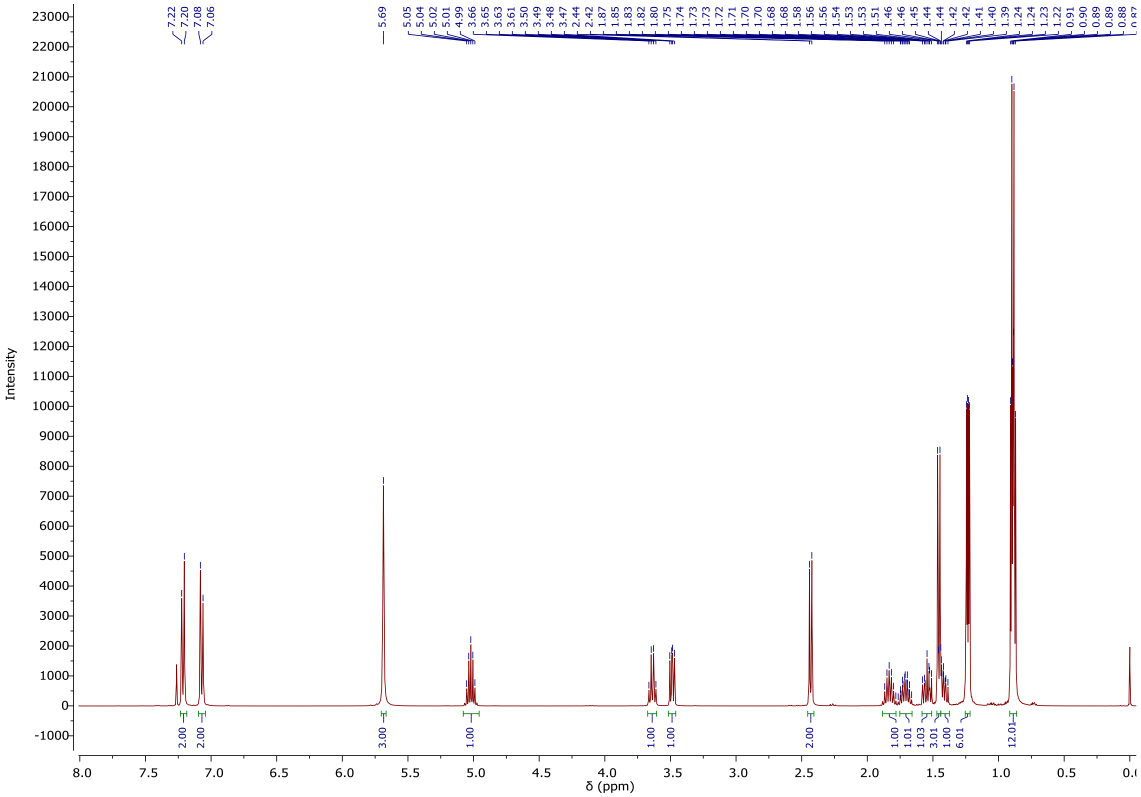

Figure S10. ^{1}H NMR spectra of [L-LeuOiPr][IBU].

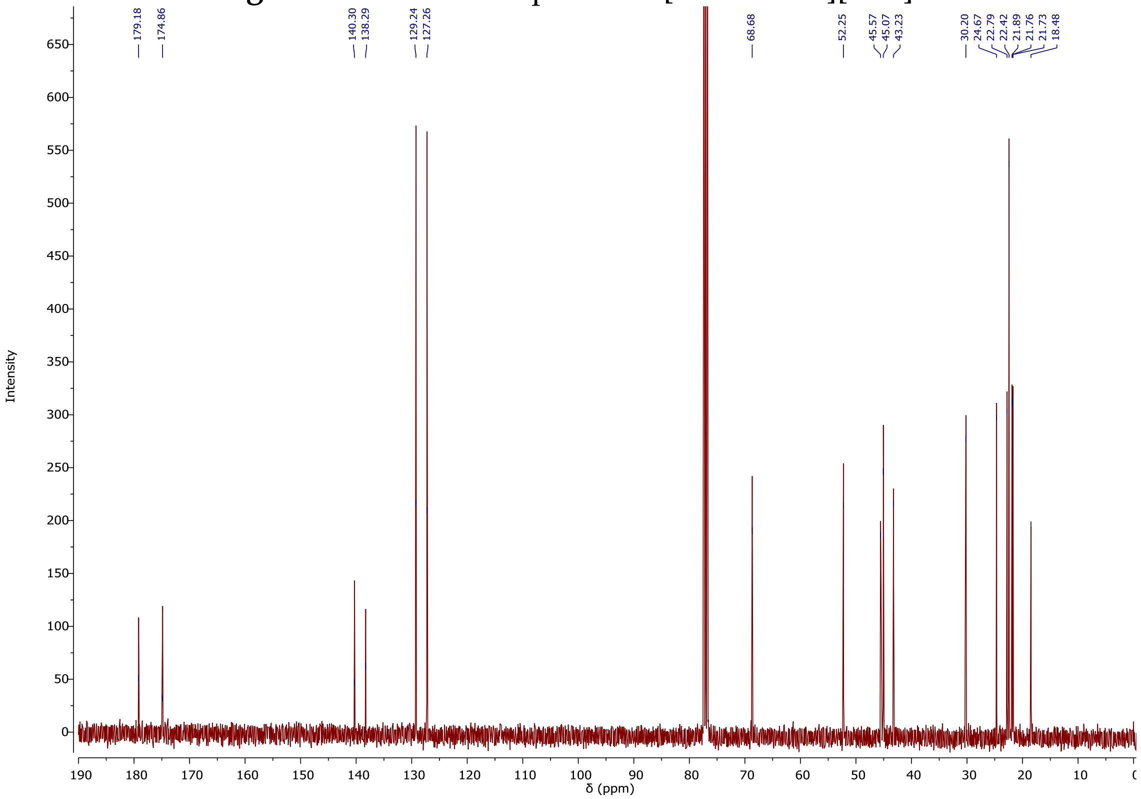

Figure S11. ^{13}C NMR spectra of [L-LeuOiPr][IBU].

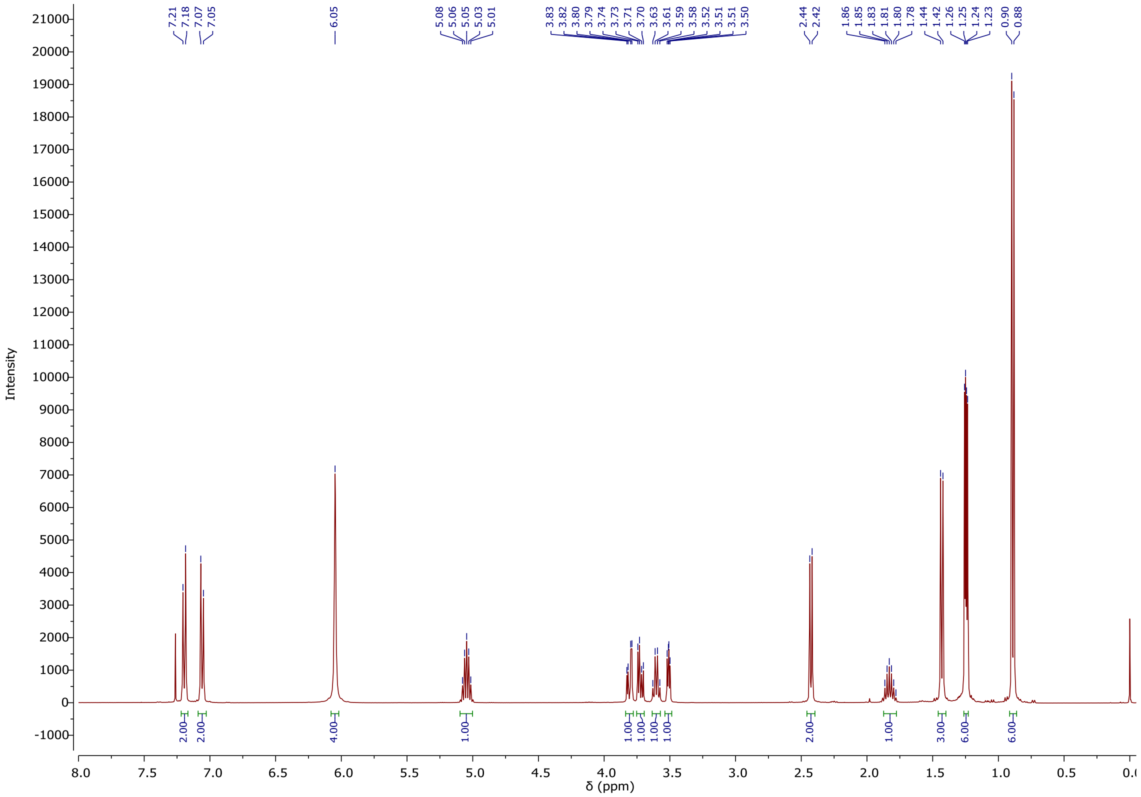

Figure S12. ^{1}H NMR spectra of [L-SerOiPr][IBU].

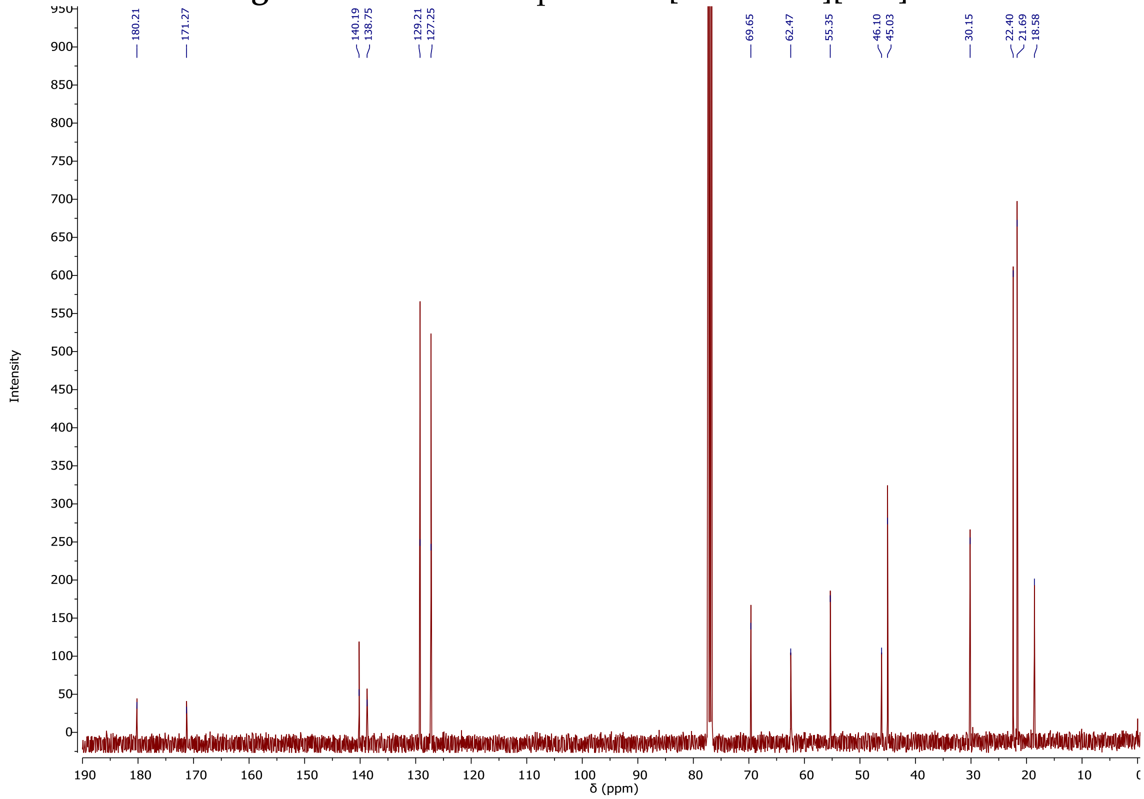

Figure S13. ^{13}C NMR spectra of [L-SerOiPr][IBU].

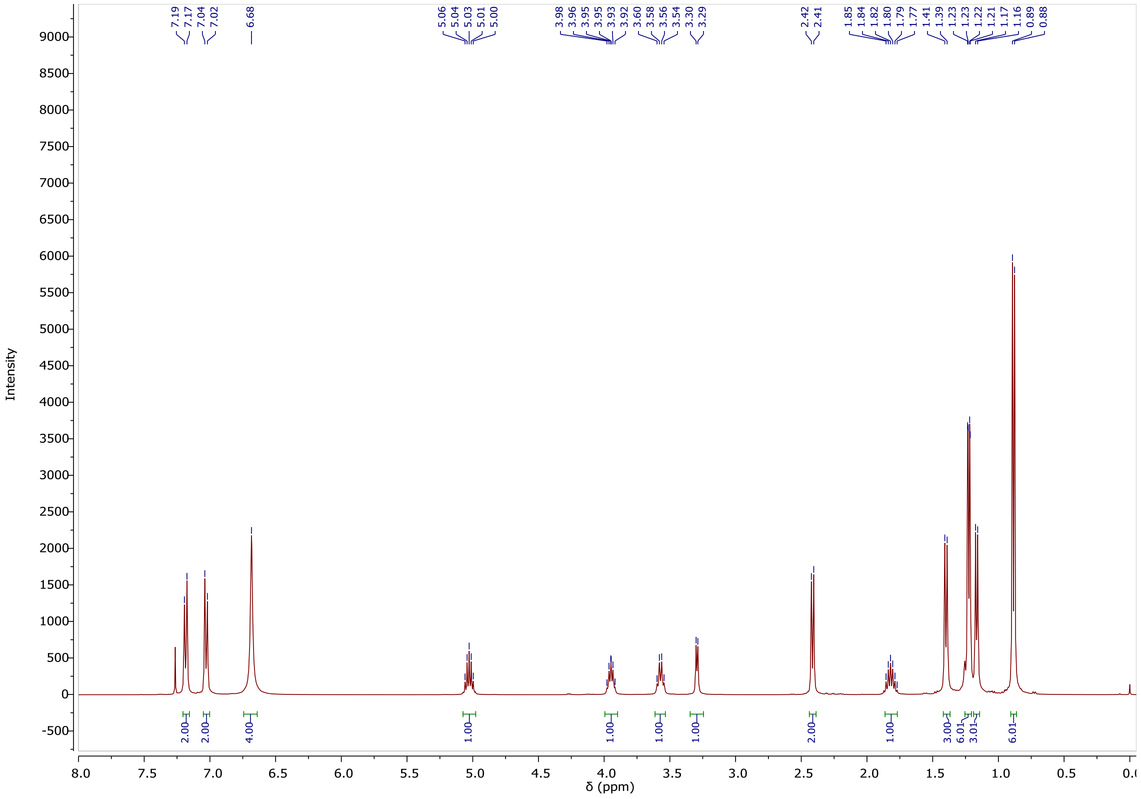

Figure S14. ^{1}H NMR spectra of [L-ThrOiPr][IBU].

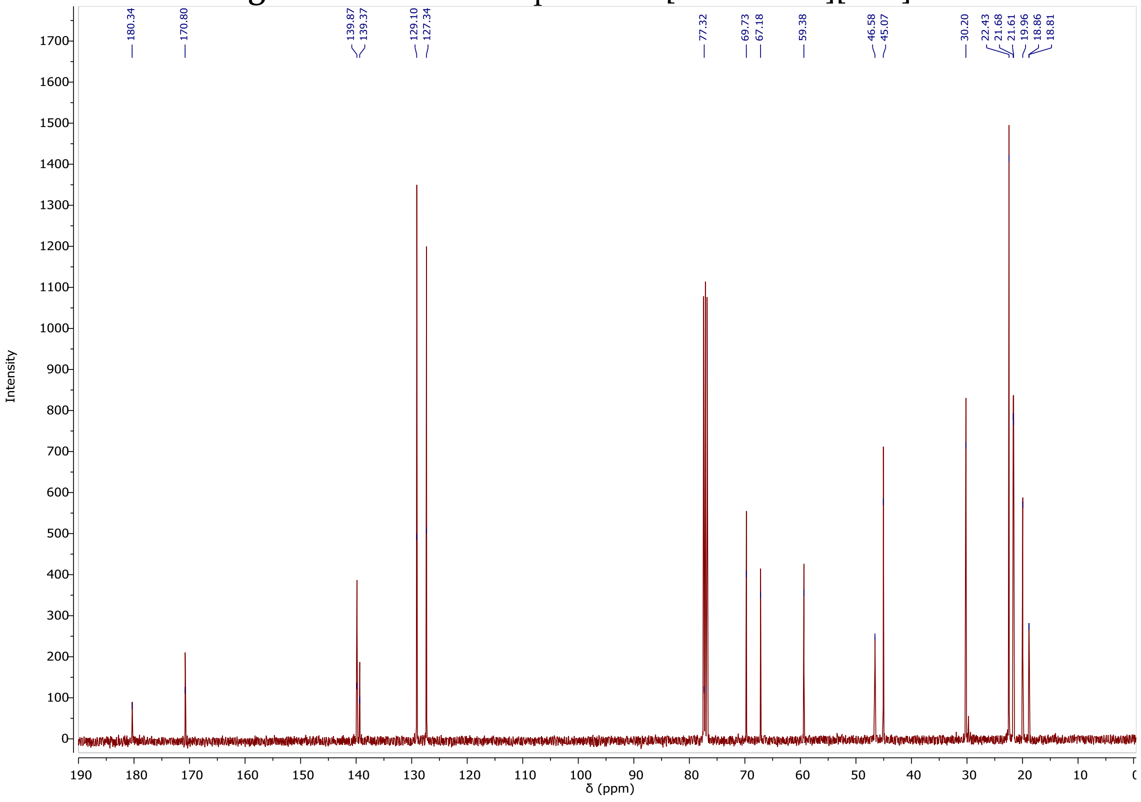

Figure S15. ^{13}C NMR spectra of [L-ThrOiPr][IBU].

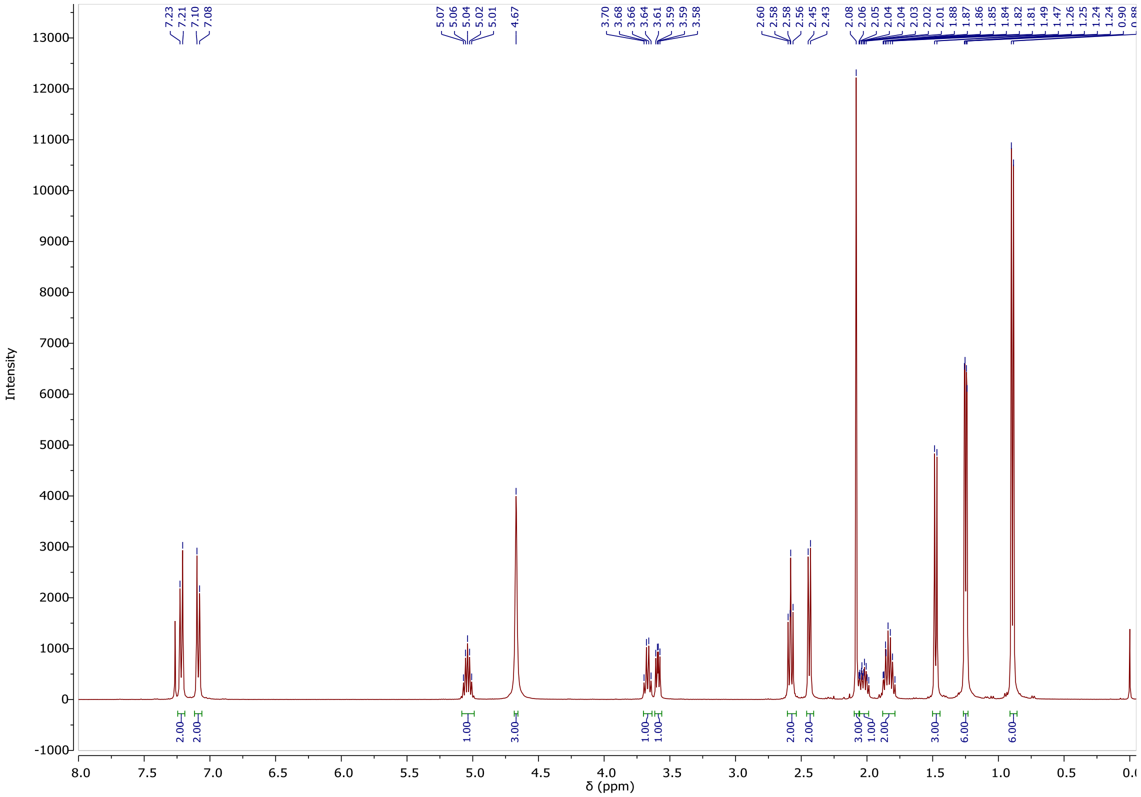

Figure S16. ^{1}H NMR spectra of [L-MetOiPr][IBU].

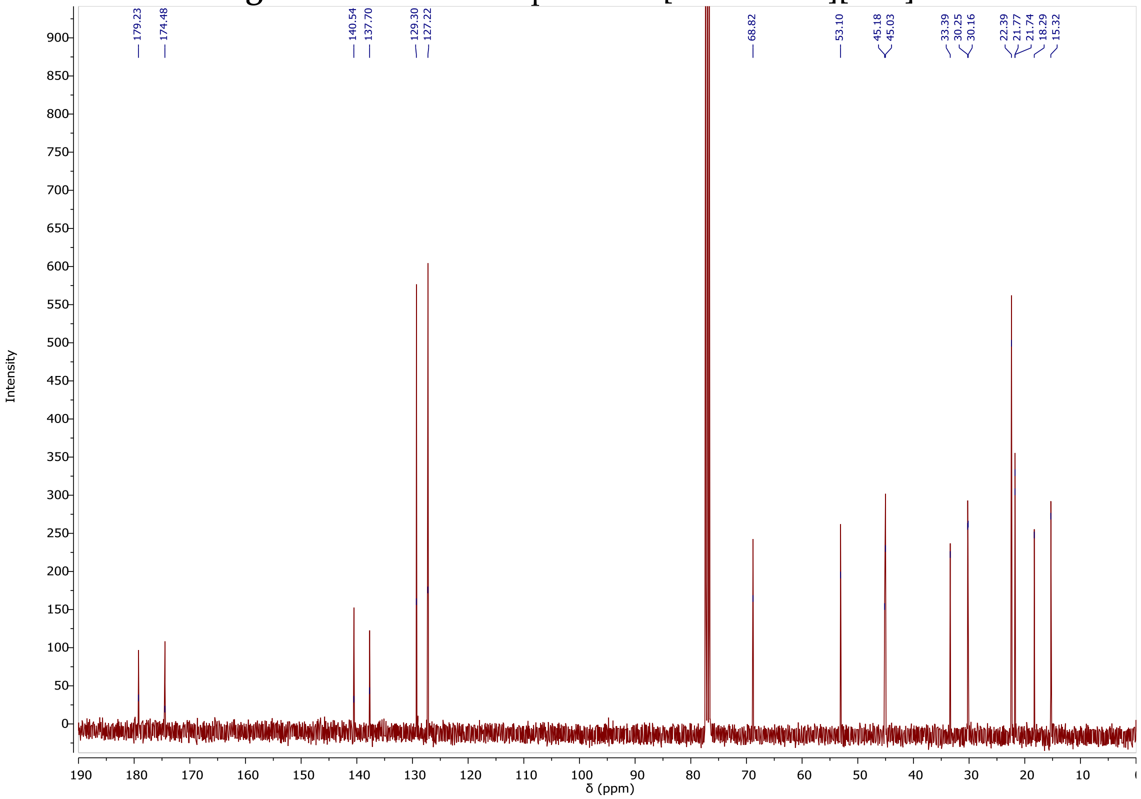

Figure S17. ^{13}C NMR spectra of [L-MetOiPr][IBU].

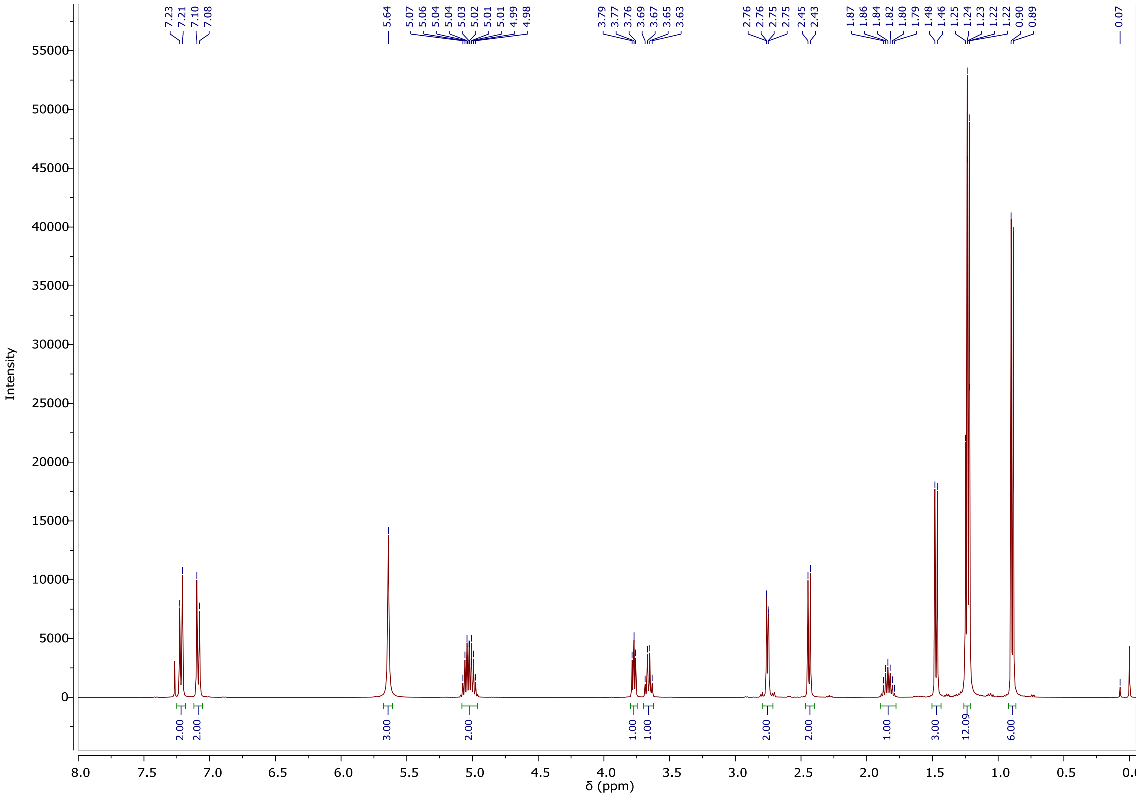

Figure S18. ^{1}H NMR spectra of [L-Asp(OiPr)$_2$][IBU].

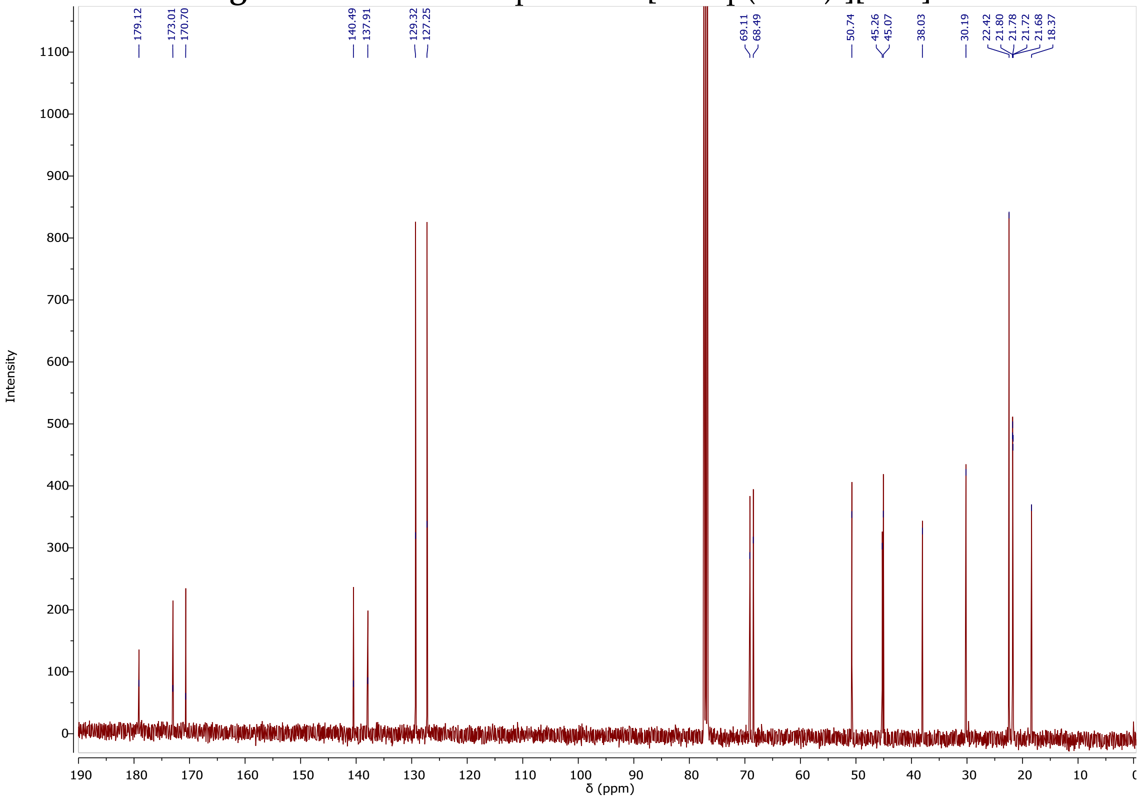

Figure S19. ^{13}C NMR spectra of [L-Asp(OiPr)$_2$][IBU].

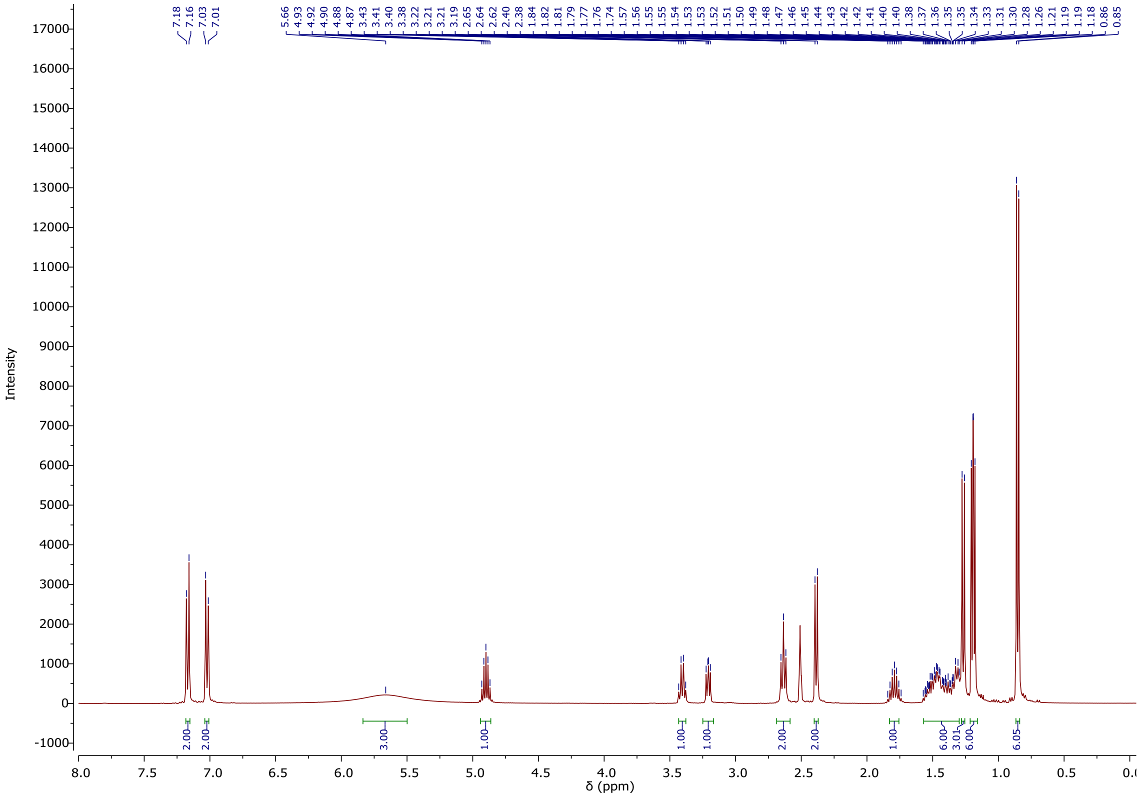

Figure S20. ^{1}H NMR spectra of [L-LysOiPr][IBU].

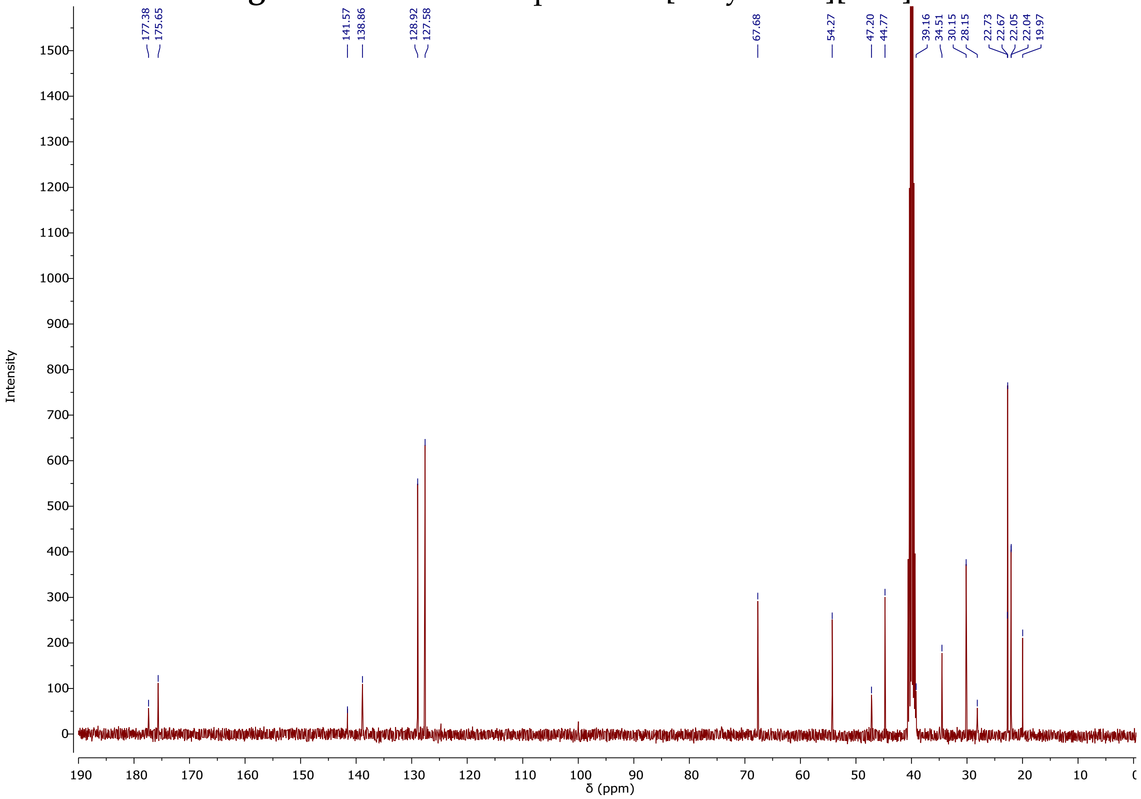

Figure S21. ^{13}C NMR spectra of [L-LysOiPr][IBU].

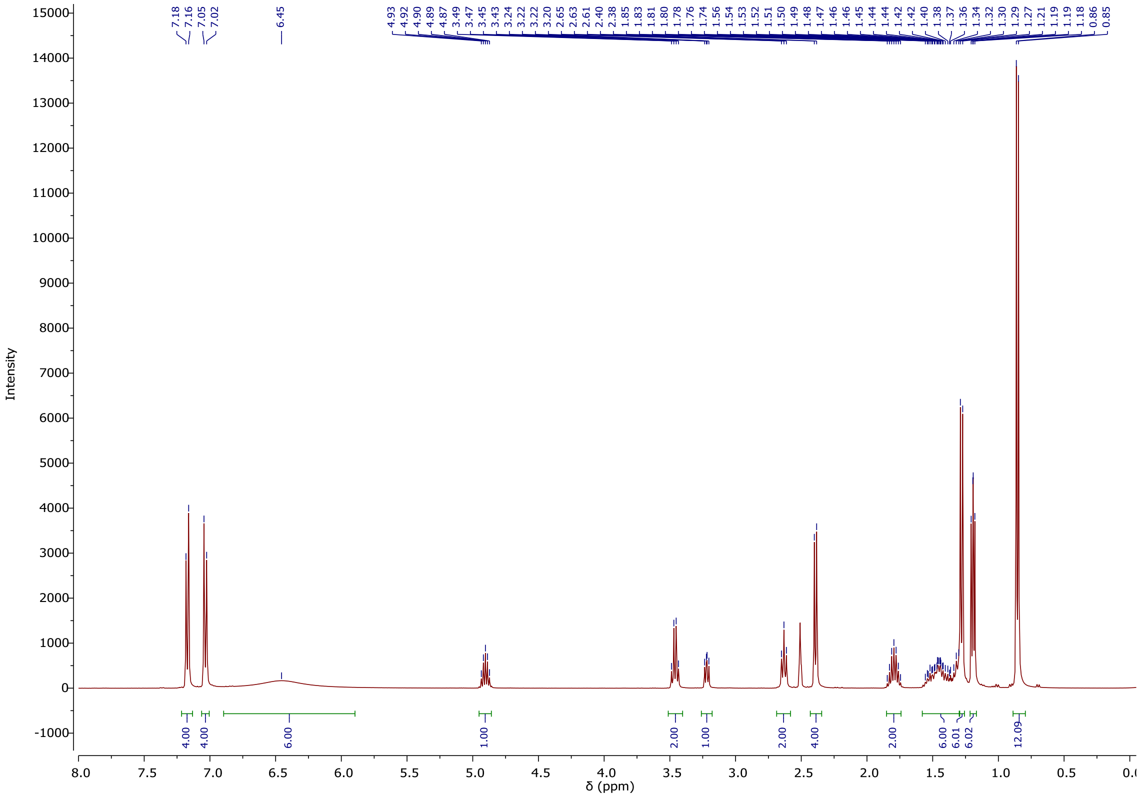

Figure S22. ^{1}H NMR spectra of [L-LysOiPr][IBU]$_2$.

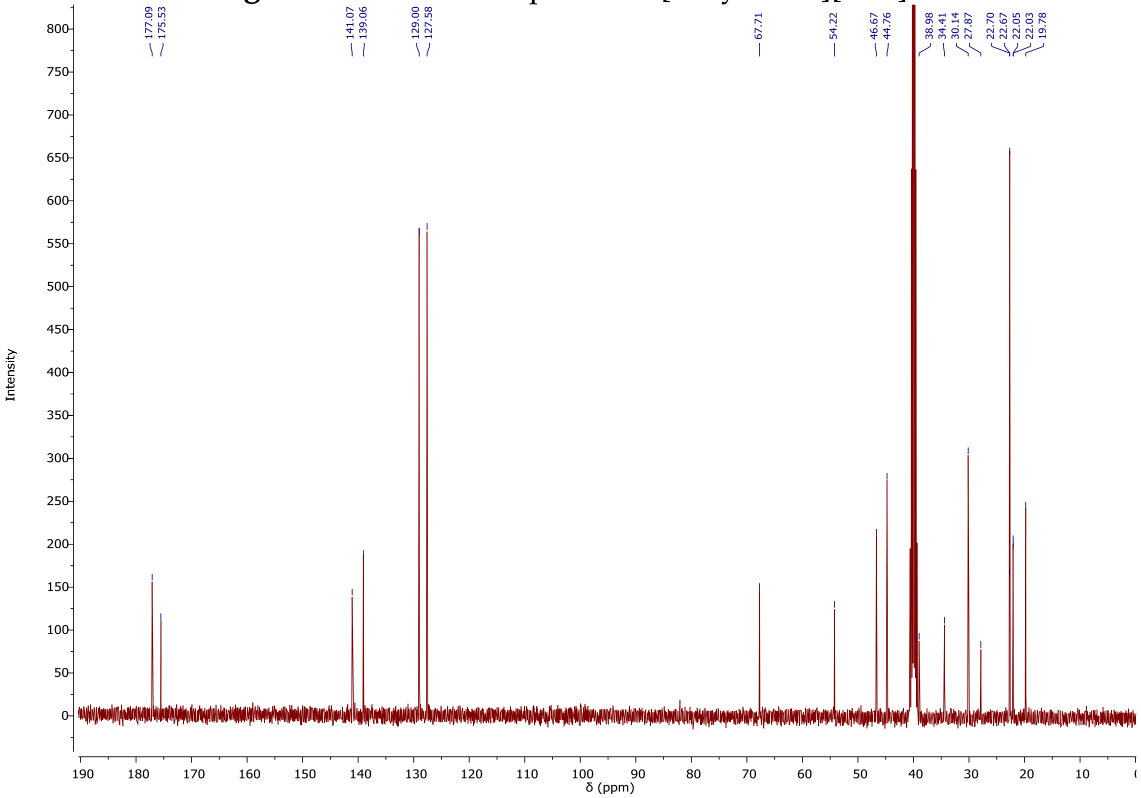

Figure S23. ^{13}C NMR spectra of [L-LysOiPr][IBU]$_2$.

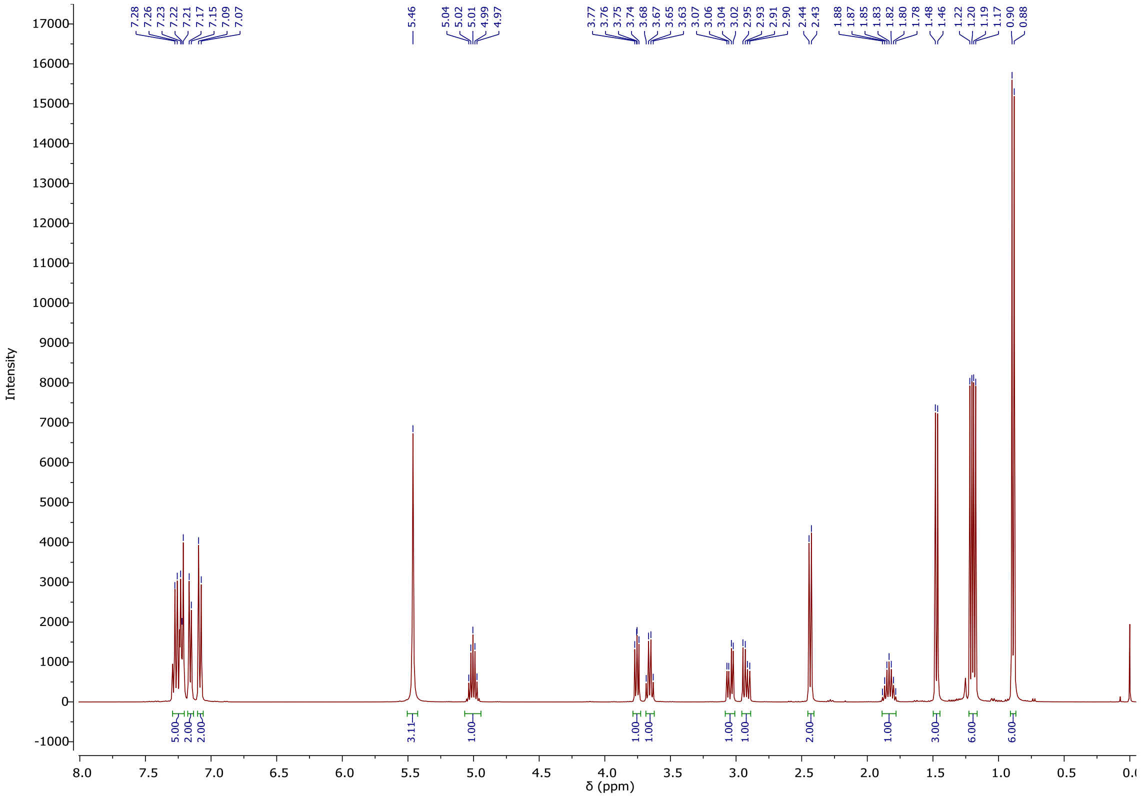

Figure S24. ^{1}H NMR spectra of [L-PheOiPr][IBU].

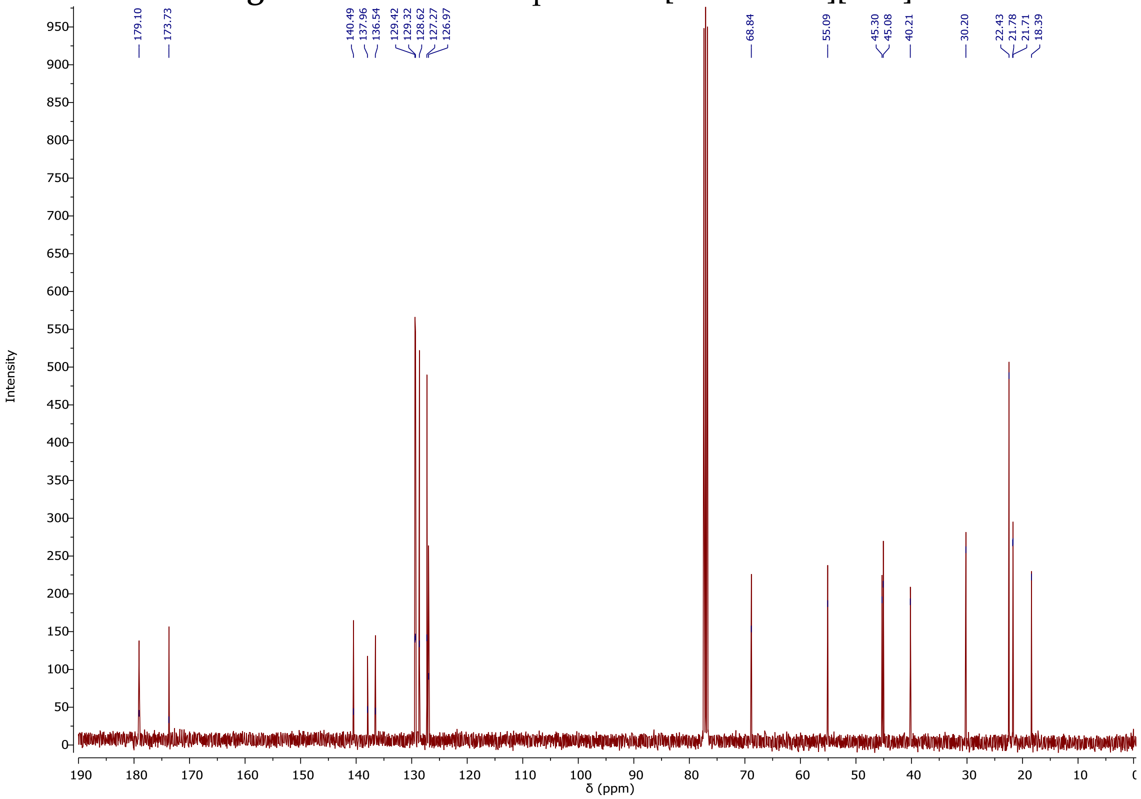

Figure S25. ^{13}C NMR spectra of [L-PheOiPr][IBU].

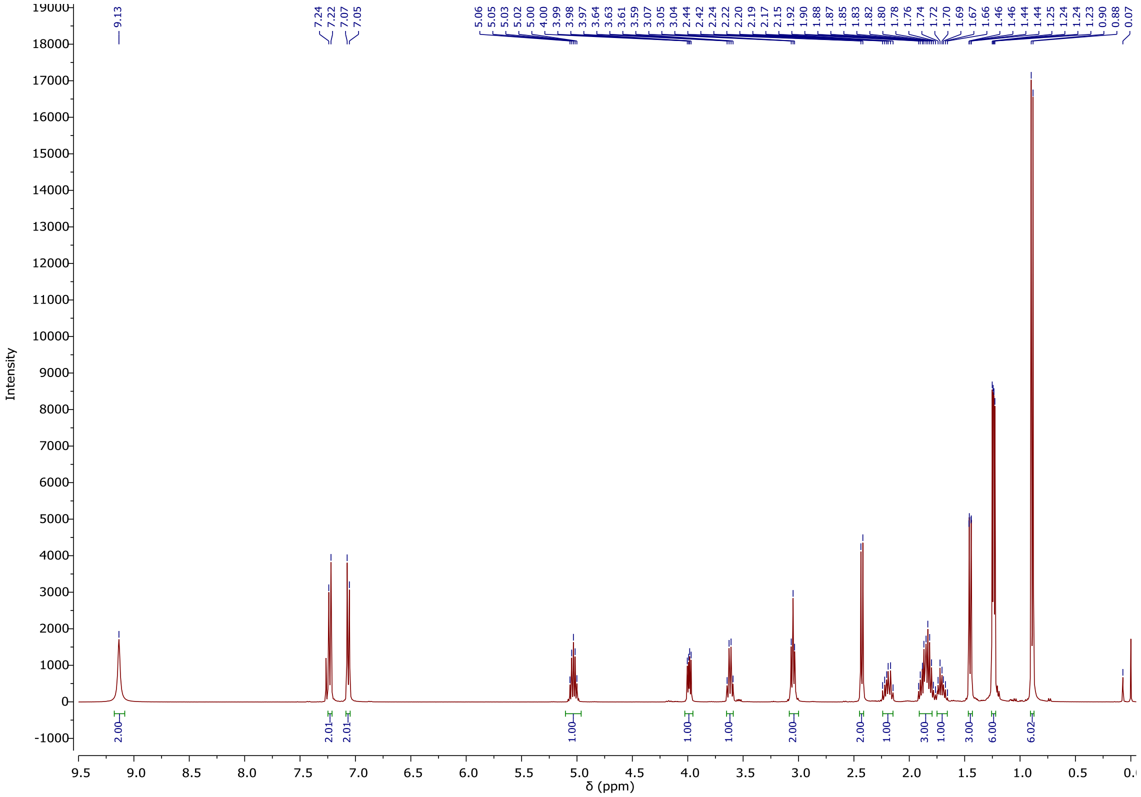

Figure S26. ^{1}H NMR spectra of [L-ProOiPr][IBU].

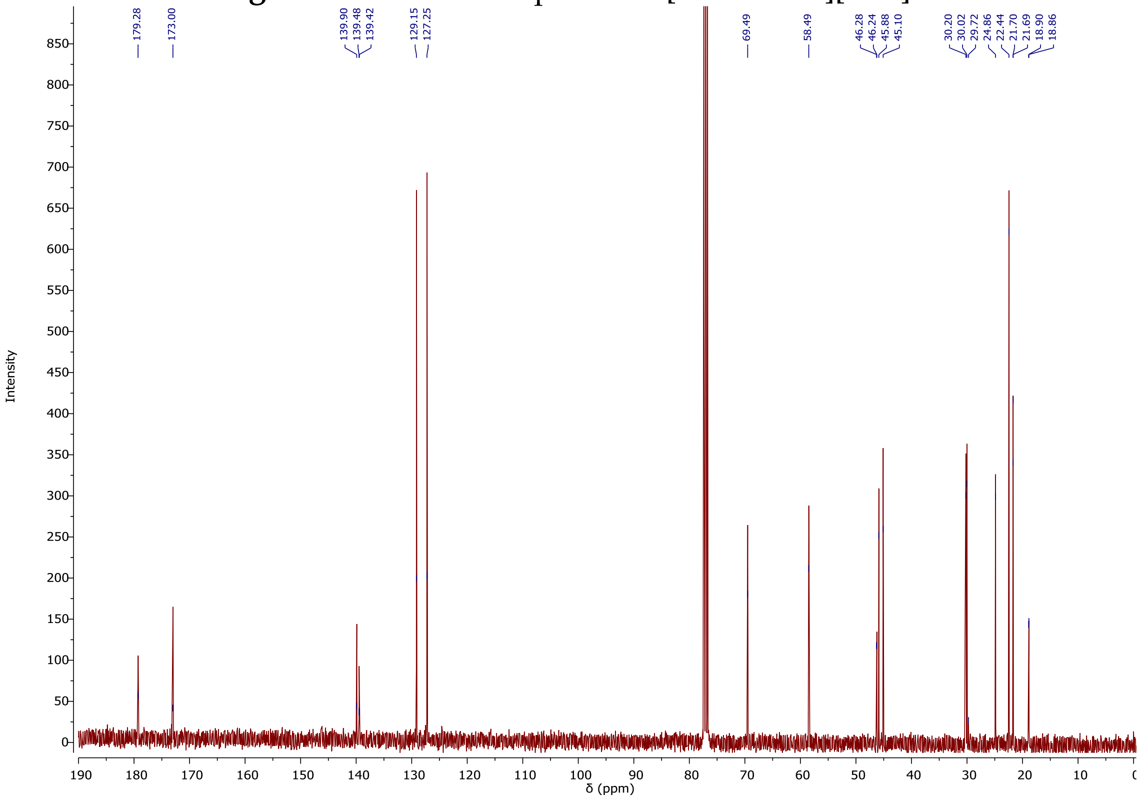

Figure S27. ^{13}C NMR spectra of [L-ProOiPr][IBU].

The ATR-FTIR spectra of [AAOiPr][IBU]

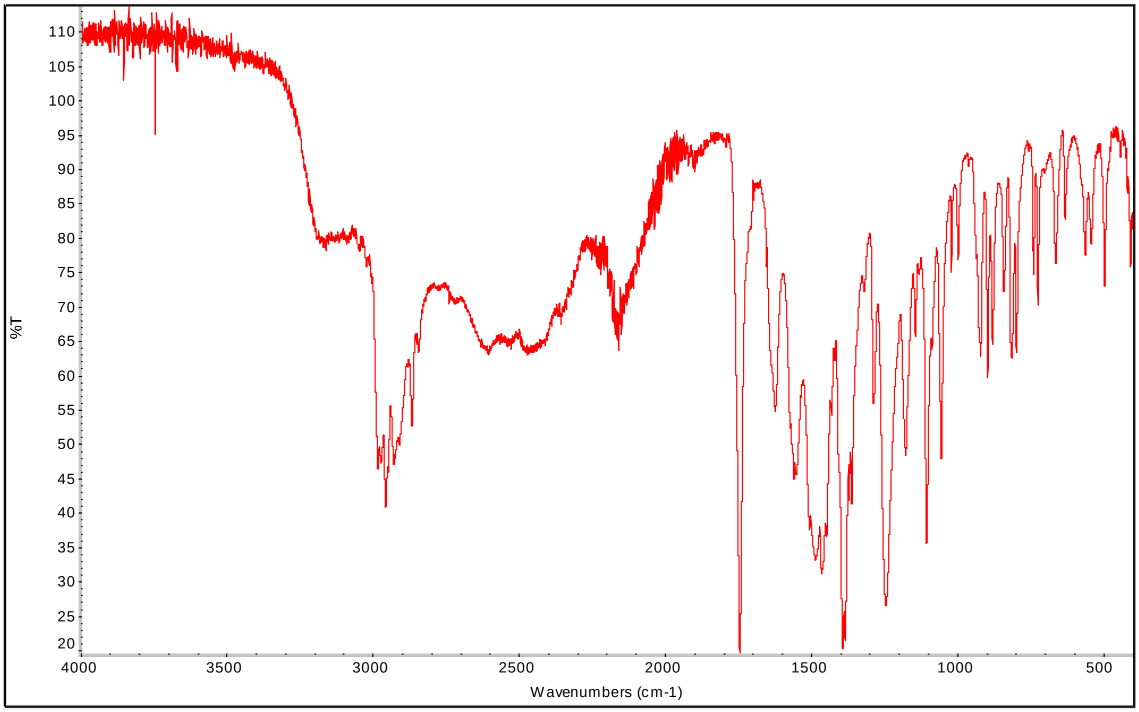

Figure S28. ATR-FTIR spectra of [GlyOiPr][IBU].

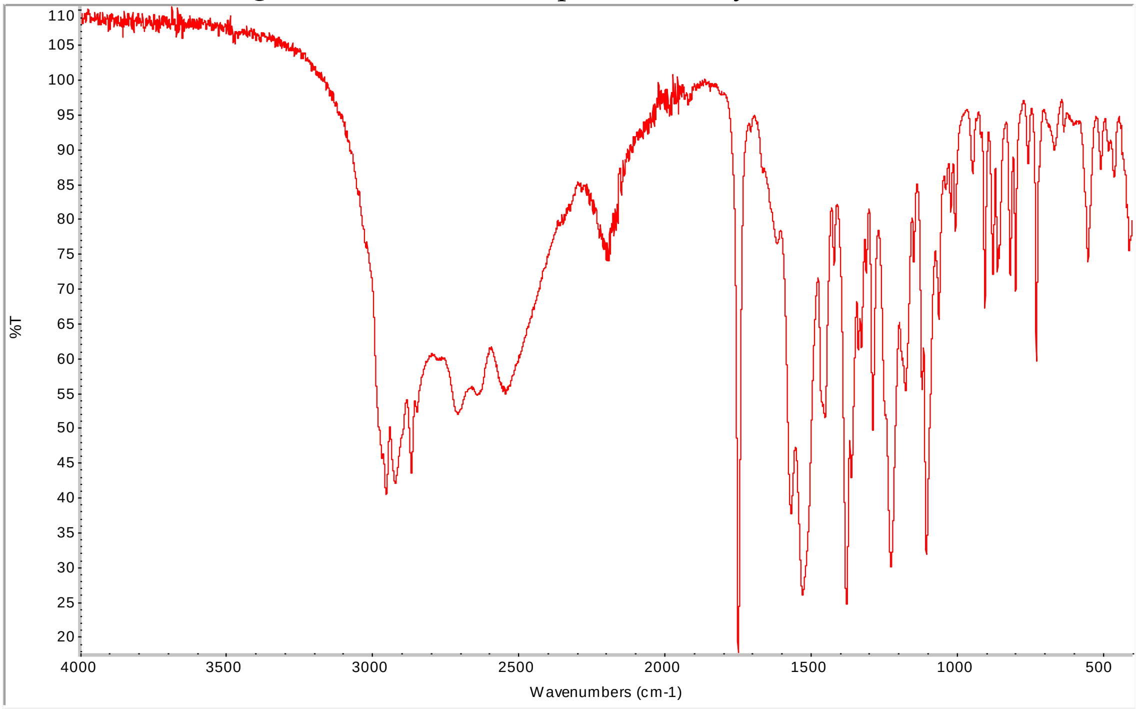

Figure S29. ATR-FTIR spectra of [L-AlaOiPr][IBU].

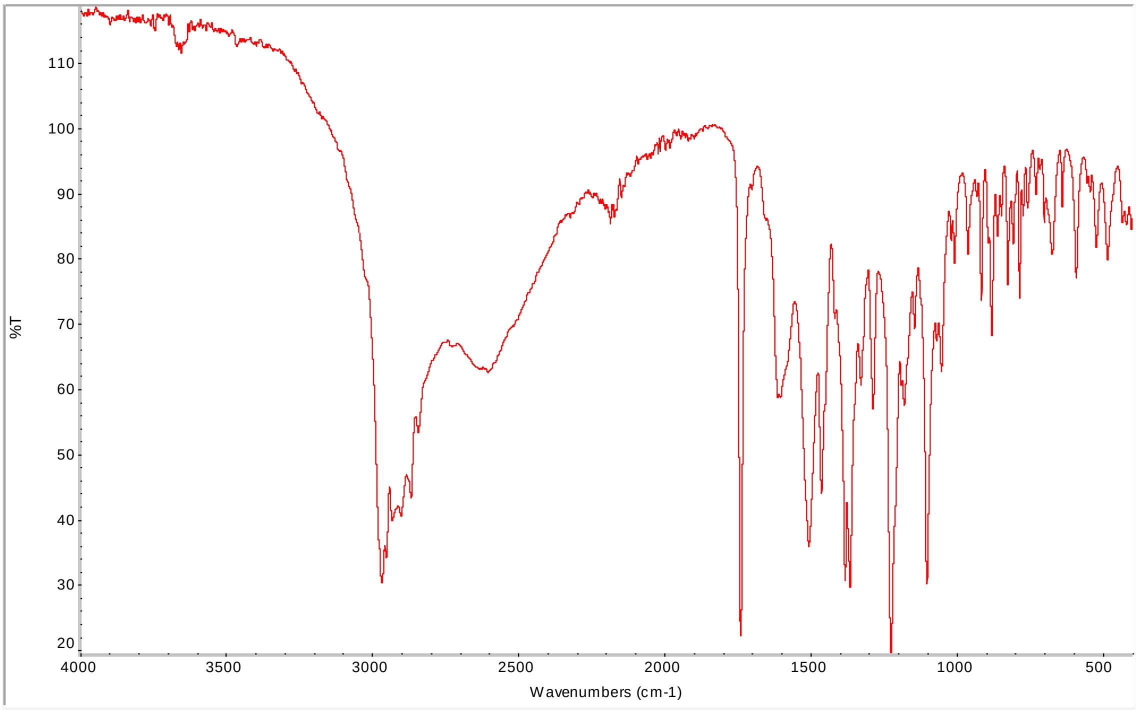

Figure S30. ATR-FTIR spectra of [L-ValOiPr][IBU].

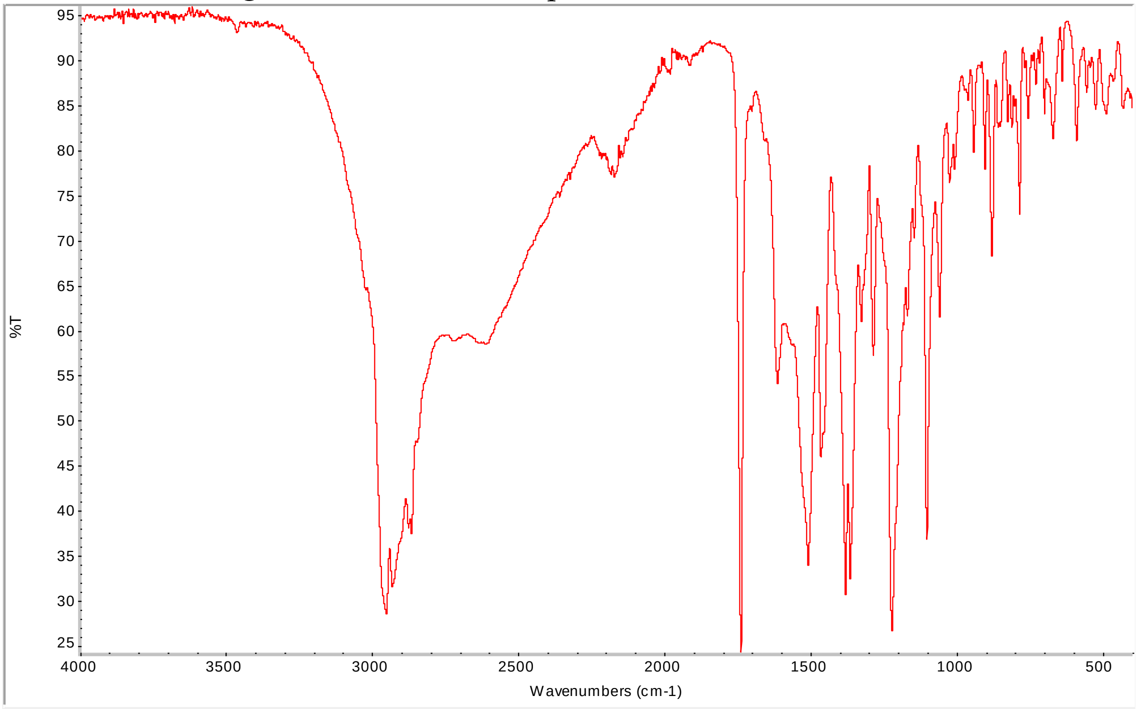

Figure S31. ATR-FTIR spectra of [L-IleOiPr][IBU].

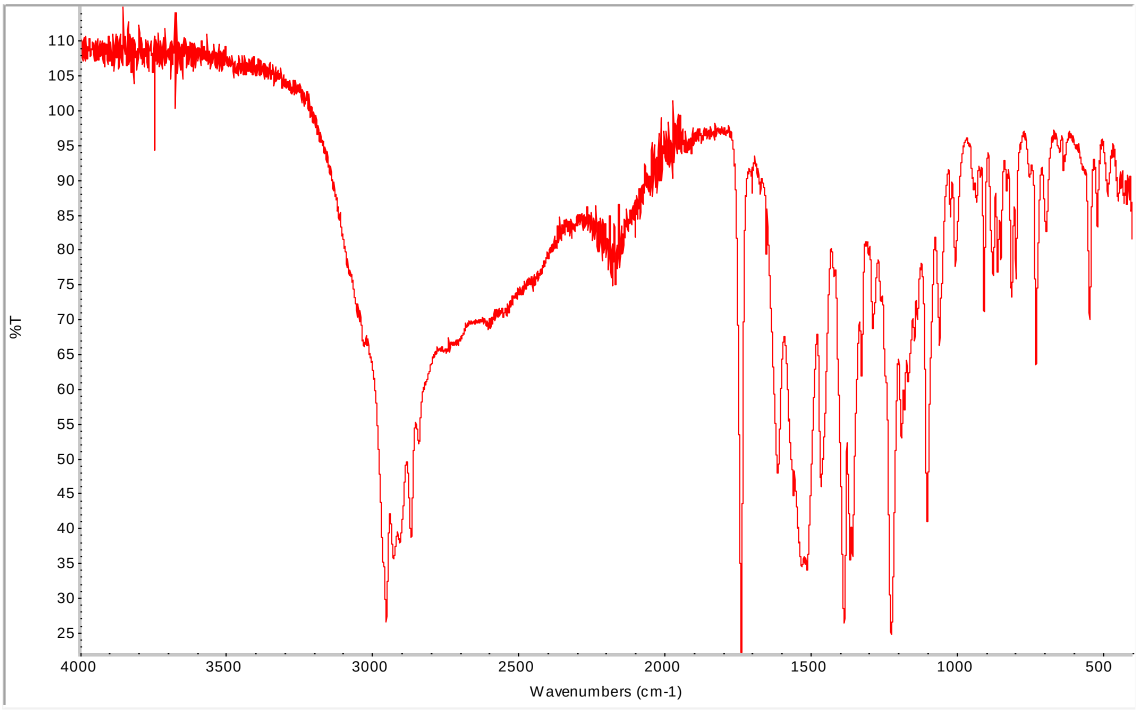

Figure S32. ATR-FTIR spectra of [L-LeuOiPr][IBU].

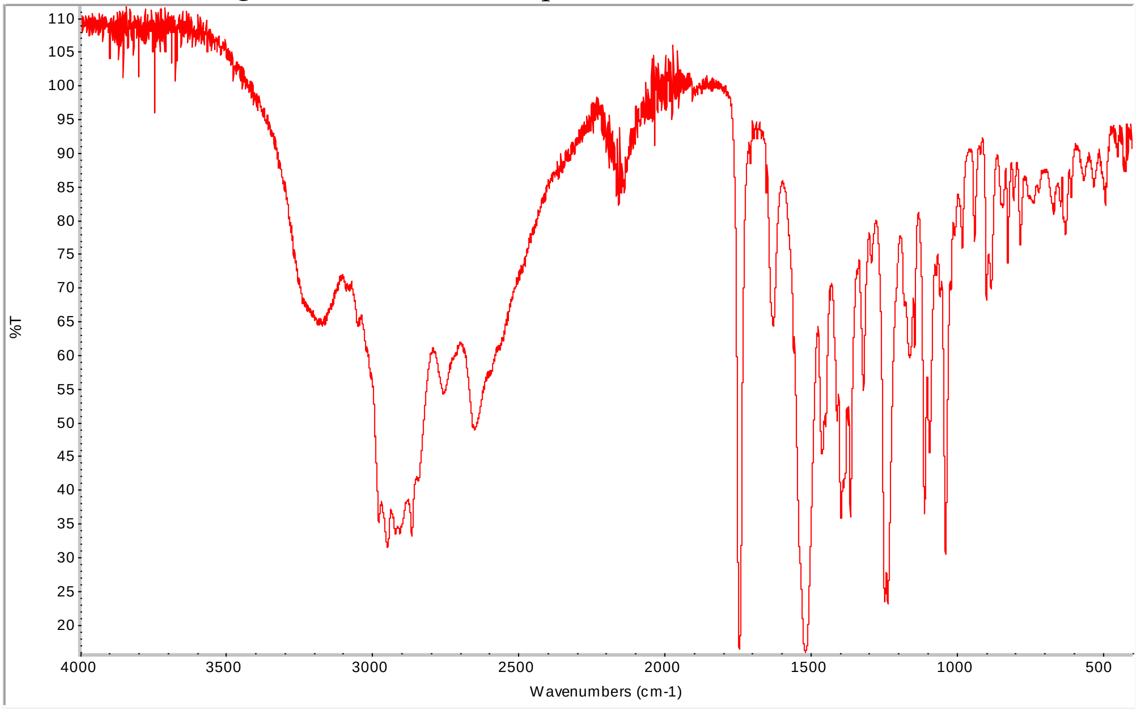

Figure S33. ATR-FTIR spectra of [L-SerOiPr][IBU].

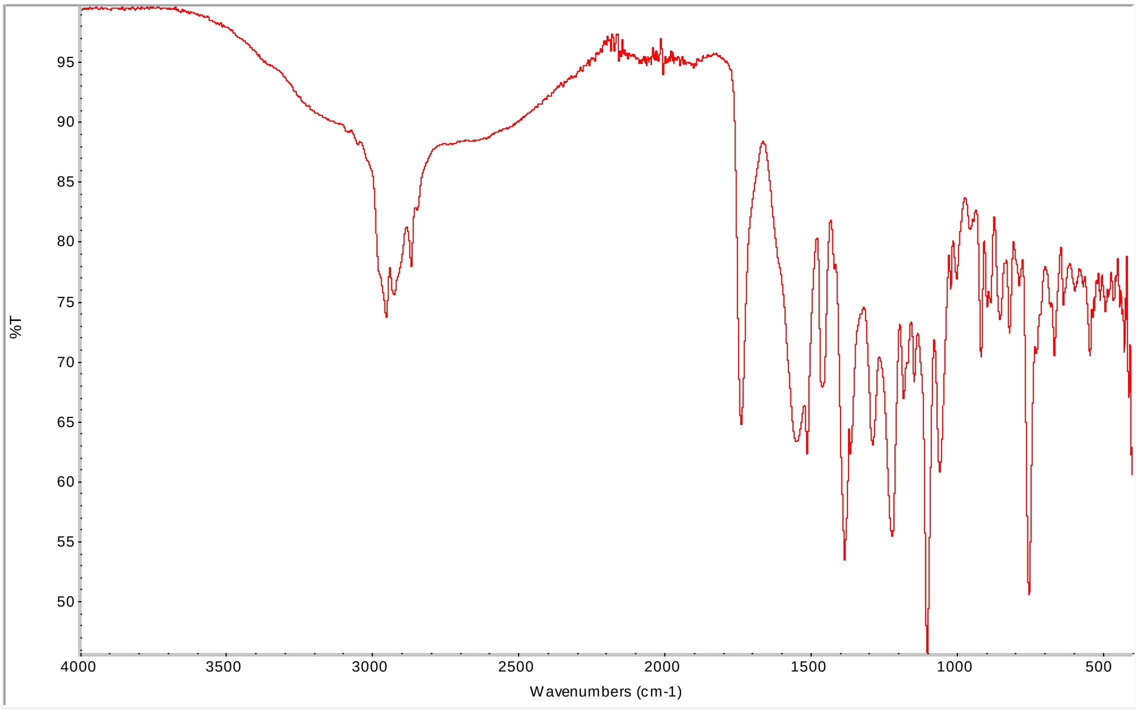

Figure S34. ATR-FTIR spectra of [L-ThrOiPr][IBU].

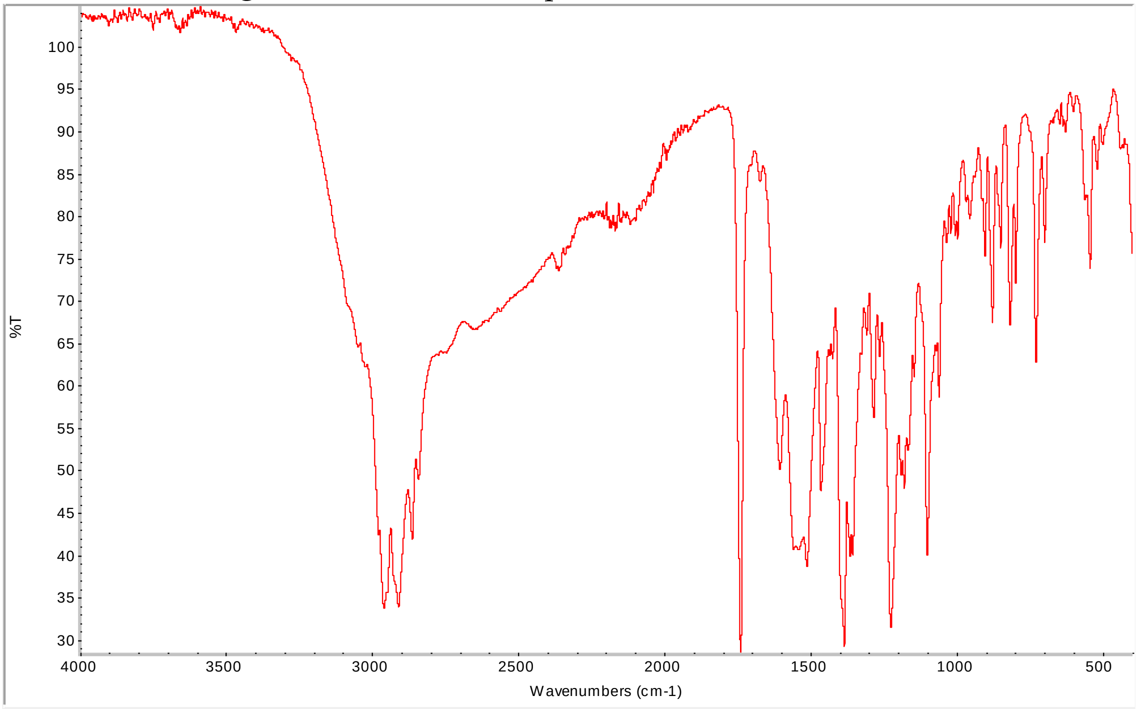

Figure S35. ATR-FTIR spectra of [L-MetOiPr][IBU].

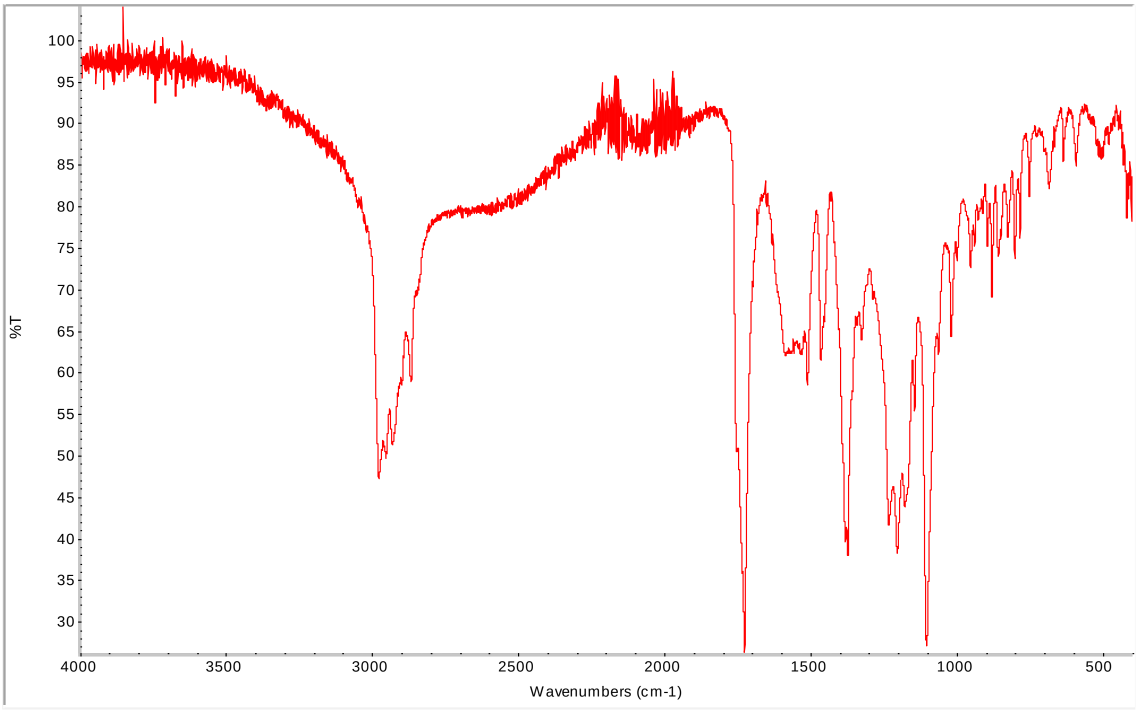

Figure S36. ATR-FTIR spectra of [L L-Asp(OiPr)2][IBU].

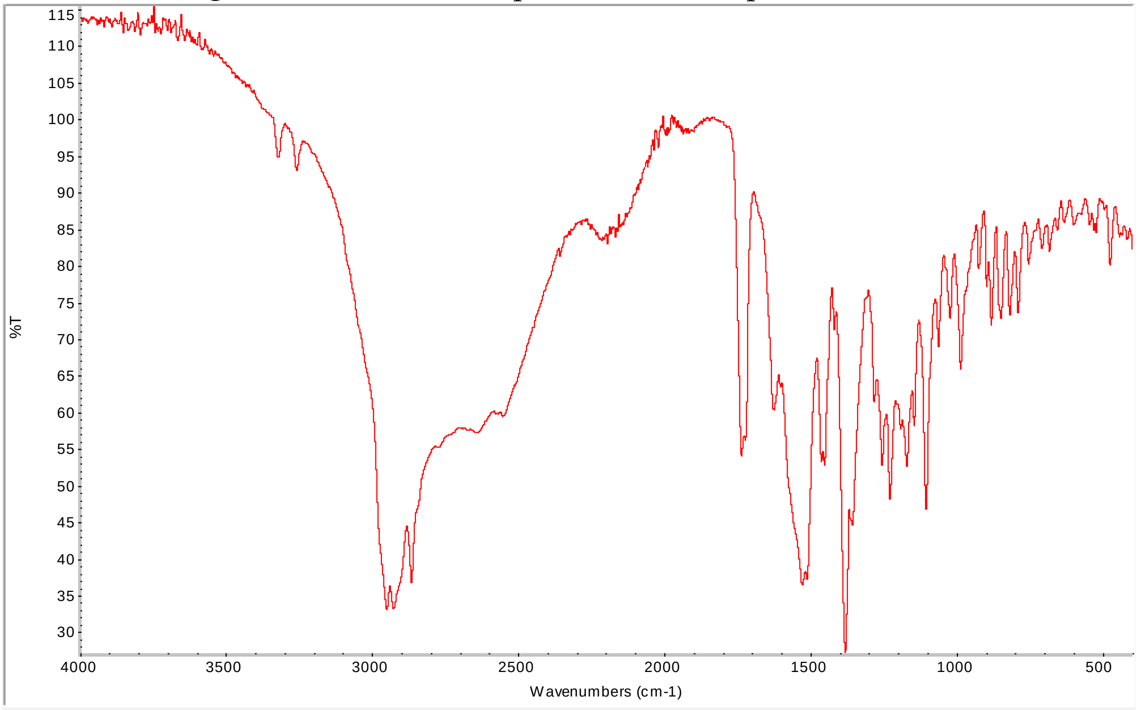

Figure S37. ATR-FTIR spectra of [L-LysOiPr][IBU].

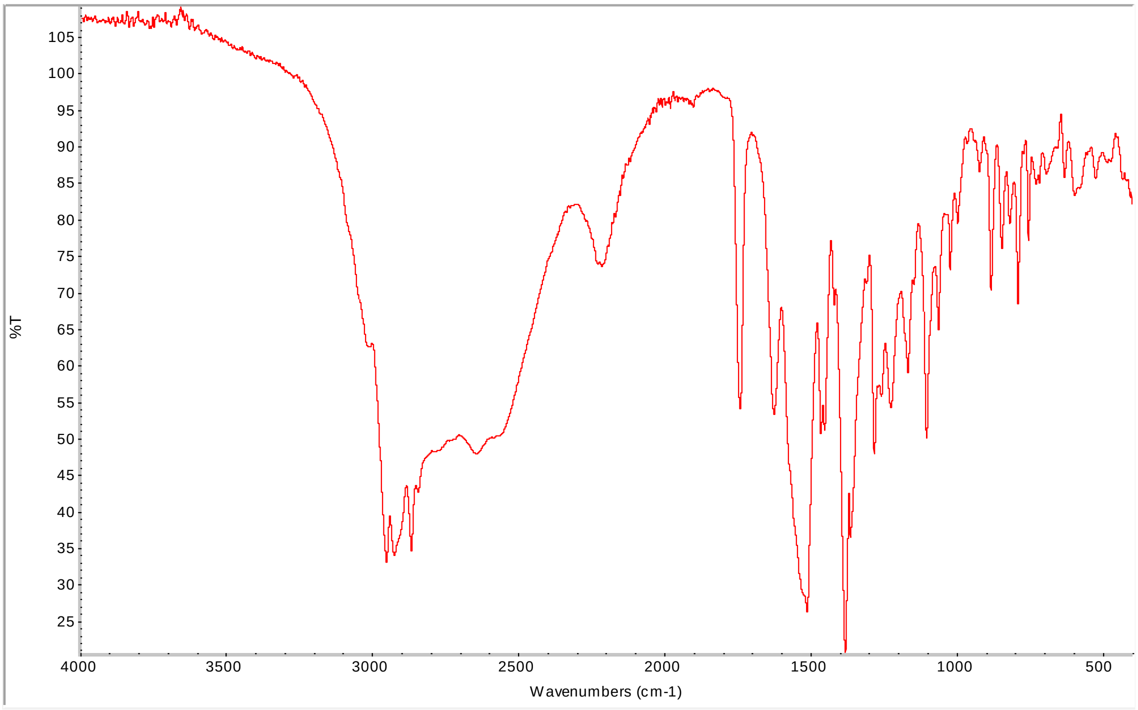

Figure S38. ATR-FTIR spectra of [L-LysOiPr][IBU]2.

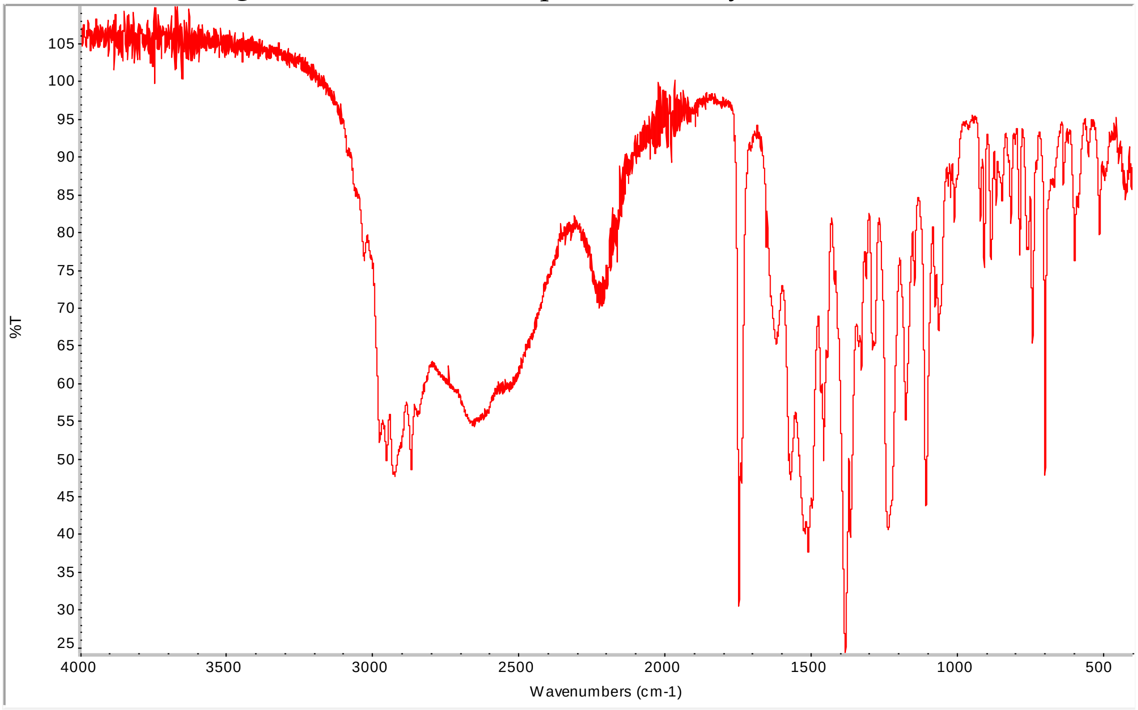

Figure S39. ATR-FTIR spectra of [L-PheOiPr][IBU].

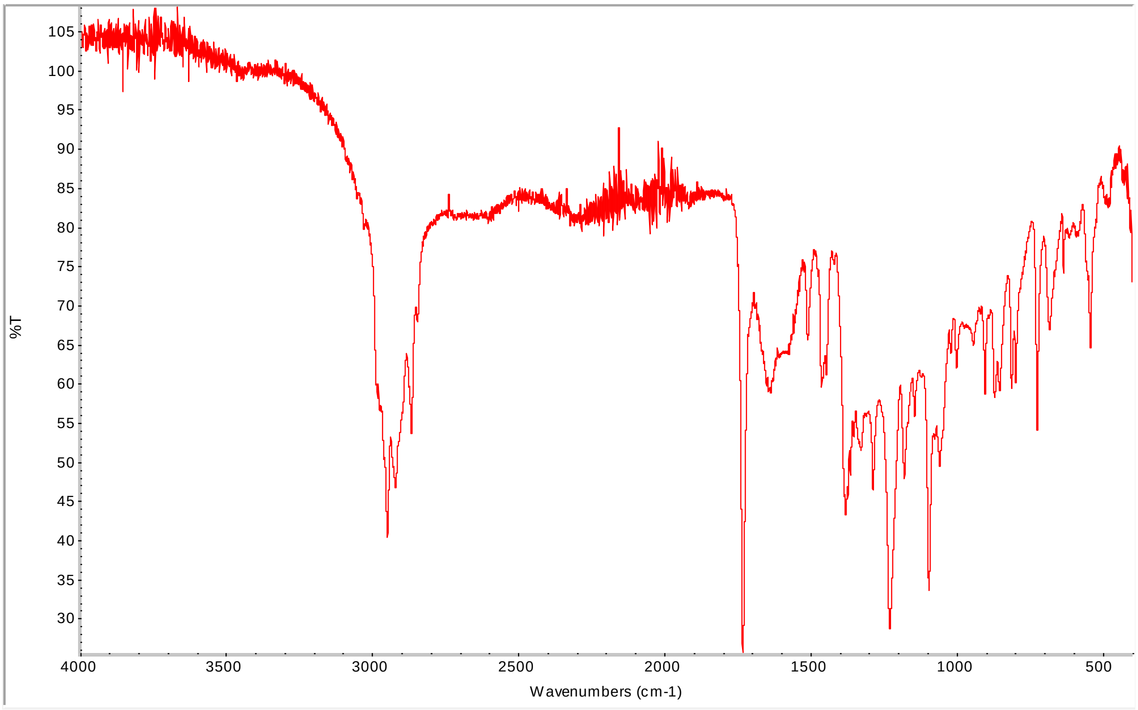

Figure S40. ATR-FTIR spectra of [L-ProOiPr][IBU].

Table S1. The antioxidant activity of the amino acid derivatives of ibuprofen and pure amino acid tested by the DPPH and ABTS method expressed as Trolox equivalent. All substances are analyzed at a concentration of 1% (calculated as active substance). All values are presented as mean ± SD, where n = 3. * Value is higher significantly from control (pure ibuprofen); different letters also mean significant differences between derivatives and pure amino acid, $\alpha = 0.050$, $p < 0.0001$.

Compounds	DPPH (mmol Trolox/dm^{-3})	ABTS (mmol Trolox/dm^{-3})
IBUPROFEN	0.145 ± 0.018	0.291 ± 0.005
[GlyOiPr][IBU]	0.209 ± 0.014 ^{a*}	0.328 ± 0.024 ^{a}
[L-AlaOiPr][IBU]	0.125 ± 0.016 ^{a}	0.262 ± 0.005 ^{a}
[L-ValOiPr][IBU]	0.168 ± 0.014 ^{a*}	0.427 ± 0.024 ^{a}
[L-IleOiPr][IBU]	0.313 ± 0.013 ^{a*}	0.987 ± 0.007 ^{a*}
[L-LeuOiPr][IBU]	0.182 ± 0.013 ^{a*}	0.561 ± 0.017 ^{a}
[L-SerOiPr][IBU]	0.413 ± 0.012 ^{a*}	1.109 ± 0.072 ^{a*}
[L-ThrOiPr][IBU]	0.264 ± 0.014 ^{a*}	0.867 ± 0.011 ^{a*}
[L-MetOiPr][IBU]	0.293 ± 0.014 ^{a*}	0.762 ± 0.026 ^{a*}
[L-Asp(OiPr)$_2$][IBU]	0.194 ± 0.013 ^{a*}	0.493 ± 0.039 ^{a*}
[L-LysOiPr][IBU]	0.029 ± 0.014 ^{a}	0.073 ± 0.011 ^{a}
[L-LysOiPr][IBU]$_2$	0.060 ± 0.015 ^{a}	0.126 ± 0.026 ^{a}
[L-PheOiPr][IBU]	0.218 ± 0.014 ^{a*}	0.684 ± 0.009 ^{a*}
[L-ProOiPr][IBU]	0.149 ± 0.015 ^{a}	0.406 ± 0.027 ^{a*}

Gly	0.081 ± 0.015 [b]	0.280 ± 0.039 [a]
L-Ala	0.095± 0.022 [b]	0.226 ± 0.046 [a]
L-Val	0.061 ± 0.004 [b]	0.147 ± 0.020 [b]
L-Ile	0.087 ± 0.016 [b]	0.327 ± 0.025 [b]
L-Leu	0.083 ± 0.026 [b]	0.310 ± 0.026 [b]
L-Ser	0.113 ± 0.003 [b]	0.431 ± 0.011 [b]
L-Thr	0.122 ± 0.027 [b]	0.298 ± 0.053 [b]
L-Met	0.114 ± 0.003 [b]	0.300 ± 0.042 [b]
L-Asp	0.093 ± 0.010 [b]	0.431 ± 0.120 [a]
L-Lys	0.016 ± 0.014 [b]	0.014 ± 0.003 [b]
L-Phe	0.125 ± 0.010 [b]	0.639 ± 0.039 [a]
L-Pro	0.082 ± 0.023 [b]	0.030 ± 0.022 [b]

Table S2. Antioxidant potential of the amino acid derivatives of ibuprofen and pure amino acid tested by the DPPH and ABTS method. Data represent the minimum, median and maximum values of three replicates. All substances are analyzed at a concentration of 1% (calculated as active substance).

	DPPH (mmol Trolox/dm^{-3})			ABTS (mmol Trolox/dm^{-3})		
Compounds	Minimum	Median	Maximum	Minimum	Median	Maximum
IBUPROFEN	0.124	0.152	0.157	0.285	0.293	0.293
[GlyOiPr][IBU]	0.194	0.212	0.221	0.302	0.332	0.349
[L-AlaOiPr][IBU]	0.106	0.134	0.134	0.259	0.259	0.267
[L-ValOiPr][IBU]	0.152	0.174	0.177	0.401	0.431	0.448
[L-IleOiPr][IBU]	0.297	0.319	0.321	0.978	0.991	0.991
[L-LeuOiPr][IBU]	0.166	0.188	0.190	0.543	0.560	0.578
[L-SerOiPr][IBU]	0.398	0.418	0.421	1.025	1.1465	1.155
[L-ThrOiPr][IBU]	0.247	0.270	0.272	0.858	0.862	0.879
[L-MetOiPr][IBU]	0.277	0.300	0.301	0.733	0.767	0.784
[L-Asp(OiPr)$_2$][IBU]	0.179	0.200	0.201	0.448	0.513	0.517
[L-LysOiPr][IBU]	0.012	0.035	0.037	0.061	0.0741	0.082
[L-LysOiPr][IBU]$_2$	0.042	0.065	0.071	0.108	0.112	0.082
[L-PheOiPr][IBU]	0.202	0.224	0.227	0.677	0.681	0.694

[L-ProOiPr][IBU]	0.132	0.155	0.159	0.384	0.397	0.435
L-Gly	0.064	0.086	0.092	0.253	0.262	0.325
L-Ala	0.070	0.103	0.112	0.176	0.235	0.266
L-Val	0.057	0.060	0.065	0.127	0.145	0.167
L-Ile	0.068	0.091	0.100	0.302	0.325	0.351
L-Leu	0.052	0.095	0.100	0.280	0.325	0.325
L-Ser	0.110	0.111	0.116	0.419	0.432	0.441
L-Thr	0.090	0.136	0.140	0.253	0.284	0.35
L-Met	0.111	0.113	0.117	0.271	0.280	0.347
L-Asp	0.081	0.098	0.098	0.342	0.383	0.567
L-Lys	0.002	0.020	0.027	0.010	0.015	0.015
L-Phe	0.117	0.121	0.135	0.612	0.621	0.684
L-Pro	0.061	0.078	0.106	0.015	0.0196	0.055

A

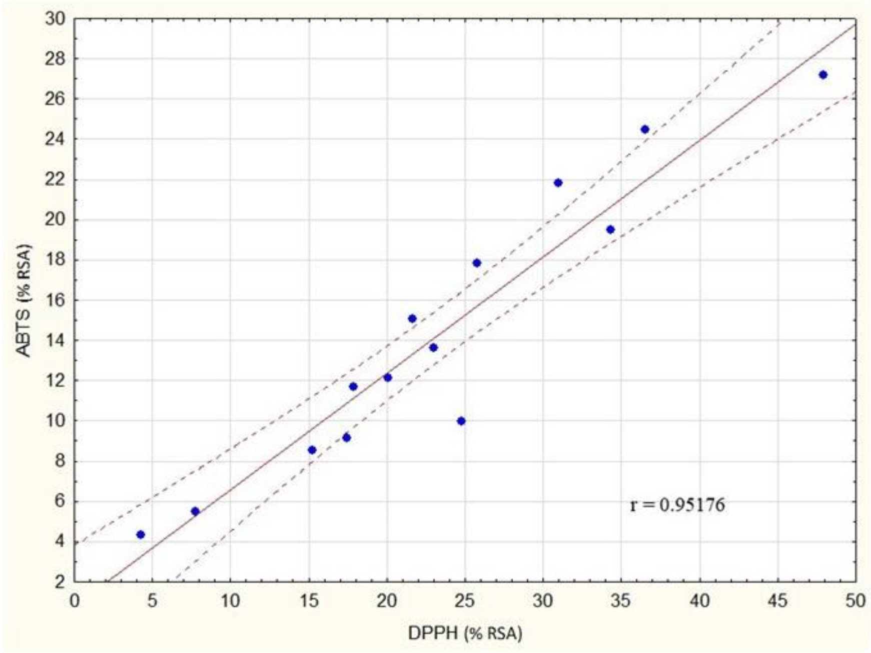

B

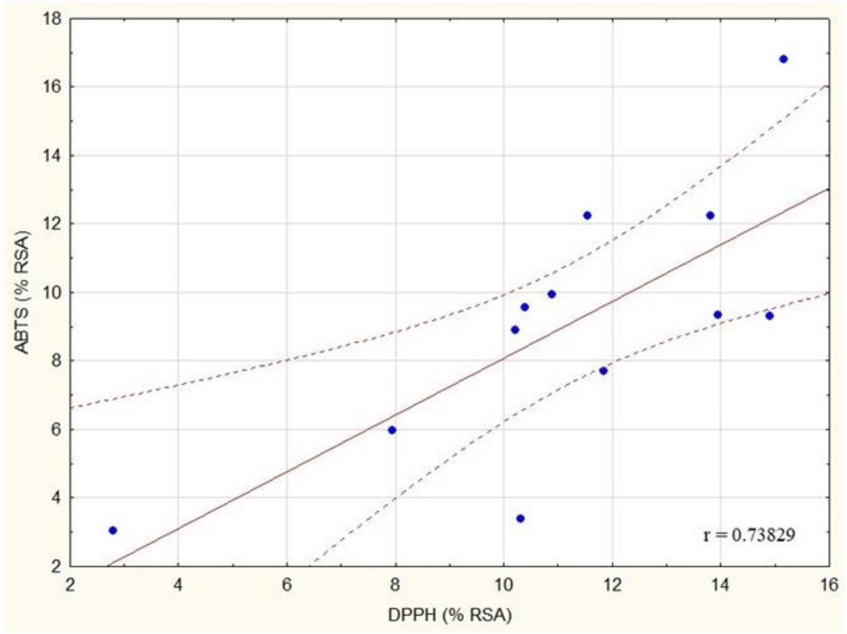

Figure S41. Correlations between the DPPH and ABTS methods for the amino acid derivatives of ibuprofen (A) and pure amino acids (B).

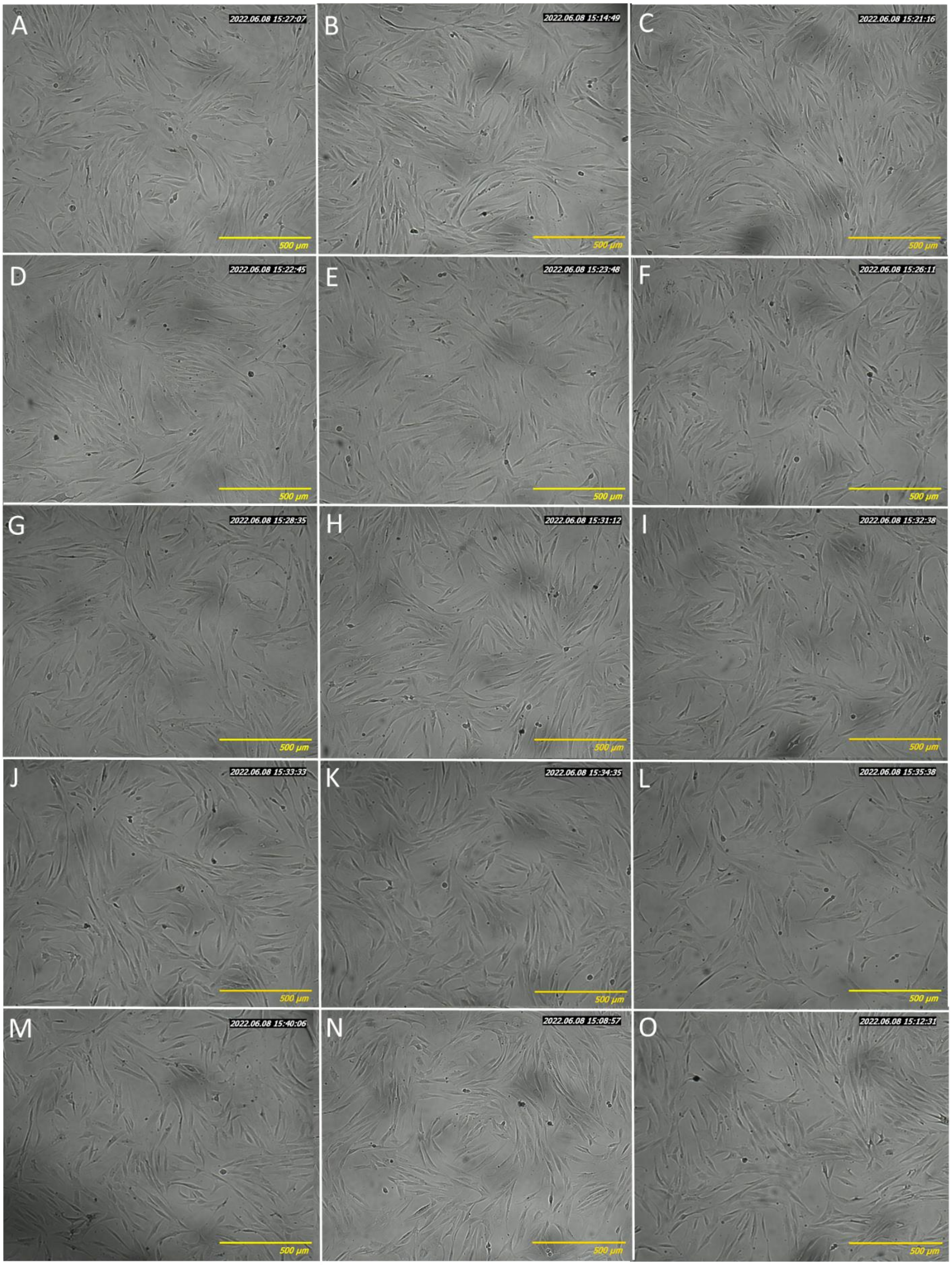
A
2022.06.08 15:27:07
500 µm
B
2022.06.08 15:14:49
500 µm
C
2022.06.08 15:21:16
500 µm
D
2022.06.08 15:22:45
500 µm
E
2022.06.08 15:23:48
500 µm
F
2022.06.08 15:26:11
500 µm
G
2022.06.08 15:28:35
500 µm
H
2022.06.08 15:31:12
500 µm
I
2022.06.08 15:32:38
500 µm
J
2022.06.08 15:33:33
500 µm
K
2022.06.08 15:34:35
500 µm
L
2022.06.08 15:35:38
500 µm
M
2022.06.08 15:40:06
500 µm
N
2022.06.08 15:08:57
500 µm
O
2022.06.08 15:12:31
500 µm

Figure S42. Representative optical microscopy images of primary human dermal fibroblasts after 24 h incubation with: (A) medium with 0.2% DMSO (negative control), (B) IBU 1000 µM (reference control), (C) [GlyOiPr][IBU] 1000 µM, (D) [L-AlaOiPr][IBU] 1000 µM, (E) [L-ValOiPr][IBU] 1000 µM, (F) [L-IleOiPr][IBU] 1000 µM, (G) [l-LeuOiPr][IBU] 500 µM, (H) [L-SerOiPr][IBU] 1000 µM, (I) [L-ThrOiPr][IBU] 1000 µM, (J) [L-MetOiPr][IBU] 1000 µM, (K) [L-Asp(OiPr)$_2$][IBU] 1000 µM, (L) [L-LysOiPr][IBU] 1000 µM, (M) [L-LysOiPr][IBU]$_2$ 500 µM, (N) [L-PheOiPr][IBU] 1000 µM, (O) [L-ProOiPr][IBU] 1000 µM.

Table S3. Physicochemical properties, lipophilicity, water solubility, pharmacokinetic analysis of ibuprofen and amino acid derivatives of ibuprofen performed using the SwissADME tool.

Molecule	MW	#Heavy atoms	#Aromatic heavy atoms	Fraction Csp3	#Rotatable bonds	#H-bond acceptors	#H-bond donors	MR	TPSA	iLOGP	XLOGP3	WLOGP
IBU	206.28	15	6	0.46	4	2	1	62.18	37.3	2.17	3.5	3.07
[GlyOiPr][IBU]	323.43	23	6	0.56	7	4	1	91.64	94.07	3.33	1.23	0.92
[L-AlaOiPr][IBU]	337.45	24	6	0.58	7	4	1	96.44	94.07	4.36	1.63	1.31
[L-ValOiPr][IBU]	365.51	26	6	0.62	8	4	1	106.06	94.07	4.64	2.59	1.94
[L-IleOiPr][IBU]	379.53	27	6	0.64	9	4	1	110.87	94.07	4.35	3	2.33
[L-LeuOiPr][IBU]	379.53	27	6	0.64	9	4	1	110.87	94.07	4.95	2.95	2.33
[L-SerOiPr][IBU]	353.45	25	6	0.58	8	5	2	97.61	114.3	3.28	0.58	0.28
[L-ThrOiPr][IBU]	367.48	26	6	0.6	8	5	2	102.41	114.3	3.57	1.06	0.67
[L-MetOiPr][IBU]	397.57	27	6	0.62	10	4	1	113.65	119.37	3.9	2.31	2.04
[L-Asp(OiPr)$_2$][IBU]	423.54	30	6	0.61	11	6	1	116.96	120.37	3.84	1.93	1.63
[L-LysOiPr][IBU]	394.55	28	6	0.64	11	5	2	113.57	120.09	4.09	1.44	1.42
[L-LysOiPr][IBU]$_2$	600.83	43	12	0.57	15	6	2	175.07	161.84	6.72	2.71	2.44
[L-PheOiPr][IBU]	413.55	30	12	0.44	9	4	1	120.93	94.07	4.77	3.26	2.53
[L-ProOiPr][IBU]	363.49	26	6	0.62	7	4	1	107.66	83.04	4.63	2.31	1.02

Table S4. Continued

Molecule	MLOGP	Silicos-IT Log P	Consensus Log P	ESOL Log S	ESOL Solubility (mg/ml)	ESOL Solubility (mol/l)	ESOL Class	Ali Log S	Ali Solubility (mg/ml)	Ali Solubility (mol/l)
IBU	3.13	3.15	3	-3.36	9.09E-02	4.41E-04	Soluble	-3.97	2.23E-02	1.08E-04
[GlyOiPr][IBU]	-1.14	3.15	1.5	-2.35	1.44E+00	4.45E-03	Soluble	-2.8	5.09E-01	1.57E-03
[L-AlaOiPr][IBU]	-0.91	3.15	1.91	-2.68	7.02E-01	2.08E-03	Soluble	-3.22	2.04E-01	6.05E-04
[L-ValOiPr][IBU]	-0.46	3.15	2.37	-3.38	1.52E-01	4.16E-04	Soluble	-4.21	2.23E-02	6.11E-05
[L-IleOiPr][IBU]	-0.24	3.15	2.52	-3.65	8.43E-02	2.22E-04	Soluble	-4.64	8.70E-03	2.29E-05
[L-LeuOiPr][IBU]	-0.24	3.15	2.63	-3.62	9.06E-02	2.39E-04	Soluble	-4.59	9.80E-03	2.58E-05
[L-SerOiPr][IBU]	-1.72	3.15	1.11	-2.05	3.18E+00	8.99E-03	Soluble	-2.55	9.88E-01	2.80E-03
[L-ThrOiPr][IBU]	-1.49	3.15	1.39	-2.43	1.37E+00	3.72E-03	Soluble	-3.05	3.26E-01	8.88E-04
[L-MetOiPr][IBU]	-0.46	3.15	2.19	-3.26	2.16E-01	5.44E-04	Soluble	-4.46	1.39E-02	3.51E-05
[L-Asp(OiPr)$_2$][IBU]	-0.91	0.72	1.44	-3.1	3.33E-01	7.87E-04	Soluble	-4.08	3.51E-02	8.28E-05
[L-LysOiPr][IBU]	-1.05	3.15	1.81	-2.63	9.34E-01	2.37E-03	Soluble	-3.57	1.07E-01	2.71E-04
[L-LysOiPr][IBU]$_2$	-3.27	3.15	2.35	-4.49	1.95E-02	3.24E-05	Moderately soluble	-5.76	1.04E-03	1.73E-06
[L-PheOiPr][IBU]	0.16	3.15	2.77	-4.16	2.86E-02	6.92E-05	Moderately soluble	-4.91	5.09E-03	1.23E-05
[L-ProOiPr][IBU]	-0.85	3.15	2.05	-3.26	2.01E-01	5.52E-04	Soluble	-3.69	7.39E-02	2.03E-04

Table S5. Continued

Molecule	Ali Class	Silicos-IT LogSw	Silicos-IT Solubility (mg/ml)	Silicos-IT Solubility (mol/l)	Silicos-IT class	GI absorption	BBB permeant	Pgp substrate
IBU	Soluble	-3.44	7.49E-02	3.63E-04	Soluble	High	Yes	No
[GlyOiPr][IBU]	Soluble	-3.44	1.17E-01	3.63E-04	Soluble	High	No	No
[L-AlaOiPr][IBU]	Soluble	-3.44	1.23E-01	3.63E-04	Soluble	High	No	No
[L-ValOiPr][IBU]	Moderately soluble	-3.44	1.33E-01	3.63E-04	Soluble	High	No	Yes
[L-IleOiPr][IBU]	Moderately soluble	-3.44	1.38E-01	3.63E-04	Soluble	High	No	Yes
[L-LeuOiPr][IBU]	Moderately soluble	-3.44	1.38E-01	3.63E-04	Soluble	High	No	Yes
[L-SerOiPr][IBU]	Soluble	-3.44	1.28E-01	3.63E-04	Soluble	High	No	No
[L-ThrOiPr][IBU]	Soluble	-3.44	1.33E-01	3.63E-04	Soluble	High	No	Yes
[L-MetOiPr][IBU]	Moderately soluble	-3.44	1.44E-01	3.63E-04	Soluble	High	No	Yes
[L-Asp(OiPr)$_2$][IBU]	Moderately soluble	-1.01	4.18E+01	9.86E-02	Soluble	High	No	No
[L-LysOiPr][IBU]	Soluble	-3.44	1.43E-01	3.63E-04	Soluble	High	No	Yes
[L-LysOiPr][IBU]$_2$	Moderately soluble	-3.44	2.18E-01	3.63E-04	Soluble	Low	No	Yes
[L-PheOiPr][IBU]	Moderately soluble	-3.44	1.50E-01	3.63E-04	Soluble	High	No	Yes
[L-ProOiPr][IBU]	Soluble	-3.44	1.32E-01	3.63E-04	Soluble	High	No	No

Table S6. Continued

Molecule	CYP1A2 inhibitor	CYP2C19 inhibitor	CYP2C9 inhibitor	CYP2D6 inhibitor	CYP3A4 inhibitor	log Kp (cm/s)	Lipinski #violations	Ghose #violations	Veber #violations	Egan #violations	Muegge #violations
IBU	No	No	No	No	No	-5.07	0	0	0	0	0
[GlyOiPr][IBU]	No	No	No	No	No	-7.4	0	0	0	0	0
[L-AlaOiPr][IBU]	No	No	No	No	No	-7.2	0	0	0	0	0
[L-ValOiPr][IBU]	No	No	No	No	No	-6.69	0	0	0	0	0
[L-IleOiPr][IBU]	No	No	No	No	No	-6.49	0	0	0	0	0
[L-LeuOiPr][IBU]	No	No	No	No	No	-6.52	0	0	0	0	0
[L-SerOiPr][IBU]	No	No	No	No	No	-8.04	0	0	0	0	0
[L-ThrOiPr][IBU]	No	No	No	No	No	-7.79	0	0	0	0	0
[L-MetOiPr][IBU]	No	No	No	No	No	-7.09	0	0	0	0	0
[L-Asp(OiPr)$_2$][IBU]	No	No	No	No	No	-7.51	0	0	1	0	0
[L-LysOiPr][IBU]	No	No	No	No	No	-7.68	0	0	1	0	0
[L-LysOiPr][IBU]$_2$	No	No	No	No	No	-8.04	1	3	2	1	2
[L-PheOiPr][IBU]	No	No	No	No	No	-6.51	0	0	0	0	0
[L-ProOiPr][IBU]	No	No	No	No	No	-6.88	0	0	0	0	0

Table S7. Continued

Molecule	Bioavailability Score	PAINS #alerts	Brenk #alerts	Leadlikeness #violations	Synthetic Accessibility
IBU	0.85	0	0	1	1.92
[GlyOiPr][IBU]	0.55	0	0	0	2.71
[L-AlaOiPr][IBU]	0.55	0	0	0	3.14
[L-ValOiPr][IBU]	0.55	0	0	2	3.46
[L-IleOiPr][IBU]	0.55	0	0	2	3.81
[L-LeuOiPr][IBU]	0.55	0	0	2	3.63
[L-SerOiPr][IBU]	0.55	0	0	2	3.37
[L-ThrOiPr][IBU]	0.55	0	0	2	3.73
[L-MetOiPr][IBU]	0.55	0	0	2	4.04
[L-Asp(OiPr)$_2$][IBU]	0.55	0	1	2	4.07
[L-LysOiPr][IBU]	0.55	0	0	2	3.79
[L-LysOiPr][IBU]$_2$	0.55	0	0	2	5.33
[L-PheOiPr][IBU]	0.55	0	0	2	3.71
[L-ProOiPr][IBU]	0.55	0	0	1	3.53
